# Field testing the transferability of behavioural science knowledge on promoting vaccinations

**DOI:** 10.1038/s41562-023-01813-4

**Published:** 2024-03-14

**Authors:** Silvia Saccardo, Hengchen Dai, Maria A. Han, Sitaram Vangala, Juyea Hoo, Jeffrey Fujimoto

**Affiliations:** 1 Department of Social and Decision Sciences, Carnegie Mellon University, Pittsburgh, PA USA; 2 Anderson School of Management, University of California Los Angeles, Los Angeles, CA USA; 3 Department of Medicine, David Geffen School of Medicine, University of California Los Angeles, Los Angeles, CA USA; 4 Department of Medicine Statistics Core, David Geffen School of Medicine, Glendon Avenue, Los Angeles, CA USA; 5 Cedars-Sinai Kerlan-Jobe Institute, Los Angeles, CA USA

**Keywords:** Human behaviour, Public health, Decision making

## Abstract

As behavioural science is increasingly adopted by organizations, there is a growing need to assess the robustness and transferability of empirical findings. Here, we investigate the transferability of insights from various sources of behavioural science knowledge to field settings. Across three pre-registered randomized controlled trials (RCTs, *N* = 314,824) involving a critical policy domain—COVID-19 booster uptake—we field tested text-based interventions that either increased vaccinations in prior field work (RCT1, NCT05586204), elevated vaccination intentions in an online study (RCT2, NCT05586178) or were favoured by scientists and non-experts (RCT3, NCT05586165). Despite repeated exposure to COVID-19 vaccination messaging in our population, reminders and psychological ownership language increased booster uptake, replicating prior findings. However, strategies deemed effective by prediction or intention surveys, such as encouraging the bundling of COVID-19 boosters and flu shots or addressing misconceptions, yielded no detectable benefits over simple reminders. These findings underscore the importance of testing interventions’ transferability to real-world settings.

## Abstract (web summary)

The authors field test the transferability of behavioural science knowledge on promoting COVID-19 booster uptake. Interventions effective in past field work improve uptake, but those deemed effective in surveys measuring intentions or predictions do not.

## Main

The successful applications of behavioural science to various policy-relevant areas^1–4^ have increasingly inspired private and public organizations, institutions and governments worldwide to seek its guidance for confronting critical challenges^5,6^. The domain of health serves a compelling illustration of this trend, with the World Health Organization integrating behavioural science into their programmes^7^, healthcare systems utilizing it to improve clinical care delivery^8^, and a rising demand for applied behavioural research to support public health measures during the recent coronavirus disease 2019 (COVID-19) pandemic^9–11^. When leveraging behavioural science to solve specific problems, scientists and practitioners may take various approaches. They may adopt interventions that have demonstrated effectiveness in prior field research. They may employ interventions that have received empirical support in hypothetical studies but are yet to be tested in the field. Or, they may develop novel interventions based on theoretical insights from the academic literature. In all cases, they must assess whether relevant scientific knowledge can effectively translate to their particular context^12^. Making these assessments is challenging, as evidenced by experts’ expectations not always aligning with what actually works in specific settings^13–17^.

To guide intervention selection, it is valuable to gather evidence about the transferability of various sources of behavioural science knowledge to field settings^18–20^. For example, assessing the transferability of interventions that have shown promise in the field is useful. This includes exploring whether strategies that were effective at inducing behaviour change at one point in time maintain their efficacy under novel, evolving circumstances. Such endeavours are often constrained by the substantial resources they require and the limited recognition for scholars who conduct replications^21,22^. Nevertheless, they are important, especially when the novelty and impact of interventions may wane over time due to repeated exposure^23,24^, and when the practical challenges of interest require repeated engagement^18^. Additionally, it is worth assessing whether interventions deemed effective in hypothetical studies and prediction surveys translate to field settings. This is because behavioural science research frequently relies on hypothetical measures, which has prompted some scholars to advocate for caution when applying behavioural science to policy^19^.

In this Article, we provide some evidence on the transferability of behavioural insights through three randomized controlled trials (RCTs) and companion surveys. Recognizing the various ways interventions are often selected in research and practice, in this research we field test interventions that are backed by various forms of scientific knowledge. Specifically, the three RCTs examine (1) interventions selected on the basis of their effectiveness in prior field experiments (RCT1), (2) interventions with promising results in online studies measuring behaviour intentions (RCT2), or (3) interventions that we developed on the basis of behavioural insights and that behavioural science experts and laypeople forecasted to be effective (RCT3). We test these interventions in the context of COVID-19 booster uptake.

Encouraging booster uptake is a timely policy challenge, given the stalled uptake of COVID-19 booster shots in many countries. For example, in the United States, while 79% of adults had completed the primary COVID-19 series as of 1 August 2023, only 20.5% of adults had received the bivalent booster^25^. Conducting RCTs in this context can help generate policy-relevant knowledge about the impact of behavioural science in promoting vaccination. Importantly, this context also offers an interesting environment for investigating whether interventions built on prior research produce consistent findings in the field, for three reasons. First, existing field evidence on COVID-19 vaccinations has focused on motivating initial vaccine acceptance, making the booster context ideal for studying whether interventions that have shown some effectiveness early on^26,27^ continue to be effective in evolving circumstances, after individuals have probably been repeatedly exposed to COVID-19 vaccine messaging. Second, the evidence about the impact of various interventions on COVID-19-related behaviour accumulated during the pandemic has mainly relied on hypothetical measures, calling for more field evaluations^10^. Third, more broadly, since the outset of the COVID-19 pandemic, behavioural scientists have offered their intuitions on what behavioural theories and findings might inform policy response^9^ and have provided evidence compilations to government agencies (for example, the Centers for Disease Control and Prevention) to help them formulate public health guidance^11^.

We conducted the RCTs in partnership with the University of California Los Angeles (UCLA) Health, a large healthcare system in California. We aimed to encourage patients who had previously completed the primary COVID-19 vaccine series to receive a bivalent booster dose. We delivered our interventions through text-based reminders. Reminders have been increasingly used as a policy tool due to their cost-effectiveness and success in numerous field evaluations^1,28,29^. In fact, text-based reminders effectively promoted initial COVID-19 vaccinations within the same population as the current sample during the early stages of vaccine distribution^26^. However, prior studies in the healthcare context have also shown that not all reminders are equally effective^16,30,31^, they may yield inconsistent findings when encouraging the same behaviour^26,32^, the size of their effects may depend on the barriers faced by the targeted audience^33,34^, and they may even have unintended negative consequences^35^. These mixed findings highlight the importance of gathering additional evidence to better understand how to design reminder interventions for improved effectiveness.

In our RCTs, we varied the presence and language of reminders. We broadly build upon the notion that behaviour change involves two pivotal stages: (1) establishing intentions to act and (2) turning these intentions into action^36–38^. To help patients turn their intentions to receive the COVID-19 bivalent booster into action, all messages in the three RCTs sought to address one common barrier to follow-through—forgetfulness—by reminding patients of their eligibility for the booster and encouraging them to get it. In the spirit of Dai et al.^26^, all interventions (except for the Doctor Recommendation Only message in the first RCT) also aimed to reduce inconvenience as another follow-through barrier by providing links to websites where patients could find convenient vaccination venues and schedule booster appointments. Alongside these components, we incorporated additional behavioural interventions to either elevate patients’ intentions to get the COVID-19 bivalent booster, further address follow-through barriers, or both. Table 1 presents the messages and condition names, which we elaborate on below.

**Table 1:** Messages tested in the three RCTs

RCT number and condition name	Message
**RCT1** **Ownership w/ Narrow Link**	UCLA Health: [Patient name], your medical records indicate that you are now eligible for the new bivalent COVID-19 booster. UCLA Health has limited booster appointments available on MyChart. To enhance your protection against COVID-19, claim your dose today by booking an appointment at CVS Pharmacy (more availability) CVS_link
**RCT1** **Ownership w/ Broad Link**	UCLA Health: [Patient name], your medical records indicate that you are now eligible for the new bivalent COVID-19 booster. UCLA Health has limited booster appointments available on MyChart. To enhance your protection against COVID-19, claim your dose today by booking an appointment at a Pharmacy nearby (more availability) General_link
**RCT1** **Doctor Recommendation Only**	UCLA Health: [Patient name], your medical records indicate that you are now eligible for the new bivalent COVID-19 booster. Doctors at UCLA Health strongly recommend that you get this updated booster, as it is designed to extend your protection against COVID-19 by targeting the most contagious, dominant variants of the virus.
**RCT1** **Doctor Recommendation & Ownership w/ Narrow Link**	UCLA Health: [Patient name], your medical records indicate that you are now eligible for the new bivalent COVID-19 booster. Doctors at UCLA Health strongly recommend that you get this updated booster, as it is designed to extend your protection against COVID-19 by targeting the most contagious, dominant variants of the virus. UCLA Health has limited booster appointments available on MyChart. Claim your dose today by booking an appointment at CVS Pharmacy (more availability) CVS_link
**RCT1** **Doctor Recommendation & Ownership w/ Broad Link**	UCLA Health: [Patient name], your medical records indicate that you are now eligible for the new bivalent COVID-19 booster. Doctors at UCLA Health strongly recommend that you get this updated booster, as it is designed to extend your protection against COVID-19 by targeting the most contagious, dominant variants of the virus. UCLA Health has limited booster appointments available on MyChart. Claim your dose today by booking an appointment at a Pharmacy nearby (more availability) General_link
**RCT2** **Simple–No Info**	UCLA Health: [Patient name], you can now get the new bivalent COVID-19 booster. UCLA Health has limited booster appointments available on MyChart. Book your appointment at a Pharmacy nearby (more availability) General_link
**RCT2** **Info–Uniqueness**	UCLA Health: [Patient name], you can now get the new bivalent COVID-19 booster, which is different from the COVID-19 vaccines you already got. The updated booster can extend your protection by targeting the most contagious, dominant variants of the virus, while strengthening your protection from earlier variants. UCLA Health has limited booster appointments available on MyChart. Book your appointment at a Pharmacy nearby (more availability) General_link
**RCT2** **Info–Eligibility Clarification**	UCLA Health: [Patient name], you can now get the new bivalent COVID-19 booster. Regardless of whether you are at high risk, received the original boosters, or previously got COVID-19, you are eligible based on your medical records, and this updated booster will strengthen your protection. UCLA Health has limited booster appointments available on MyChart. Book your appointment at a Pharmacy nearby (more availability) General_link
**RCT2** **Info–Severity**	UCLA Health: [Patient name], the chances that a healthy adult will develop severe or long-lasting COVID-19 symptoms are higher than many people realize. You can now get the new bivalent COVID-19 booster, which can effectively reduce your chance of developing severe illness and long-lasting COVID-19 symptoms. UCLA Health has limited booster appointments available on MyChart. Book your appointment at a Pharmacy nearby (more availability) General_link
**RCT2** **Consistency**	UCLA Health: [Patient name], based on your medical records, you have completed a COVID-19 vaccine primary series. Great job protecting your health. Now, you can get the new bivalent COVID-19 booster. UCLA Health has limited booster appointments available on MyChart. Book your appointment at a Pharmacy nearby (more availability) General_link
**RCT2** **Consistency & Info–Uniqueness**	UCLA Health: [Patient name], based on your medical records, you have completed a COVID-19 vaccine primary series. Great job protecting your health. Now, you can get the new bivalent COVID-19 booster, which is different from the COVID-19 vaccines you already got. The updated booster can extend your protection by targeting the most contagious, dominant variants of the virus, while strengthening your protection from earlier variants. UCLA Health has limited booster appointments available on MyChart. Book your appointment at a Pharmacy nearby (more availability) General_link
**RCT3** **Simple–Enhance Protection**	UCLA Health: [Patient name], this fall, enhance your protection against COVID-19! You can now get the bivalent COVID-19 booster. UCLA Health has limited booster appointments available on MyChart. Book your appointment at CVS Pharmacy (more availability) CVS_link
**RCT3** **Bundle–Tagging Flu Shot**	UCLA Health: [Patient name], this fall, enhance your protection against COVID-19! You can now get the bivalent COVID-19 booster. UCLA Health has limited booster appointments available on MyChart. Book your appointment at CVS Pharmacy (more availability) CVS_link; you can also protect yourself against the flu by adding the flu vaccine to your COVID-19 booster appointment.
**RCT3** **Bundle–Booster & Flu Shot**	UCLA Health: [Patient name], this fall, enhance your protection against COVID-19 and the flu! You can now save time by bundling two vaccines (the bivalent COVID-19 booster and flu vaccine) at once. UCLA Health has limited booster appointments available on MyChart. At CVS Pharmacy (more availability), you can book one appointment to get both vaccines CVS_link

This table shows the text messages used from the fourth day of the RCTs onwards. See Methods for the minor changes we made to text messages on the fourth day and the reason. To conserve space, we have replaced the exact Uniform Resource Locators (URLs) in the messages with short names. See exact URLs in Supplementary Methods, see Supplementary Table 10 for the correspondence between condition name in Main and condition name in the pre-registrations, and see ref. ^50^ for how the actual messages exactly looked like on a phone as well as the messages sent during the first three days of the RCTs.

**Fig. 1:**
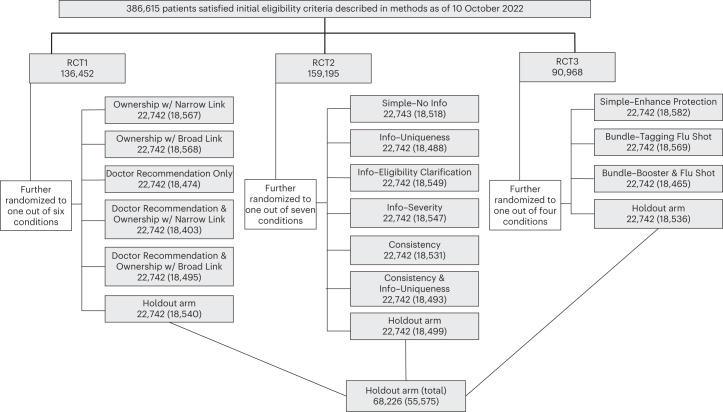
Randomization of patients into condition across three RCTs. This figure shows the randomization of patients into three RCTs and specific experimental conditions. In each box corresponding to a specific condition, the first number refers to the number of patients who were randomly assigned to the corresponding condition, and the number in parentheses refers to the number of patients who satisfied the pre-registered inclusion/exclusion criteria for data analysis (as described in Methods) and thus were used in data analysis. Patients who were randomly assigned to the holdout arm in each RCT were collapsed into one aggregate holdout arm for final data analysis.

Across three RCTs, eligible patients (*N* = 386,615) were randomly assigned to either one of 14 message conditions or the holdout condition (standard of care). We designed each RCT to be self-contained and address distinct research questions. Following the megastudy approach^39^, we concurrently conducted the three RCTs, which enabled us to test the relative effectiveness of interventions across RCTs, assess the transferability of interventions based on different knowledge sources, and accelerate scientific progress in identifying the best strategies for promoting vaccinations. Figure 1 depicts the randomization process.

In the first RCT (*N* = 136,452; ref. ^40^) we delivered intention- and action-oriented interventions based on strategies that previous field experiments identified as effective for promoting initial COVID-19 vaccinations. In a prior RCT^26^ involving the same population as the current RCTs, reminders that induced a sense of psychological ownership by asking patients to ‘claim their dose’ were the best-performing reminders, leading to higher vaccine uptake than basic reminders that simply prompted action with a link to schedule appointments. To examine whether the effectiveness of reminders with ownership language would translate to booster uptake, our first RCT included messages containing the ‘claim your dose’ language (Ownership w/ Narrow Link and Ownership w/ Broad Link arms).

The first RCT also capitalized on evidence from an experiment in the Czech Republic where correcting misconceptions about doctors’ endorsement of the COVID-19 vaccine increased the uptake of initial COVID-19 doses^27^. Considering this evidence, along with our own survey results indicating a positive correlation between perceived doctor endorsements of bivalent boosters and booster uptake intentions in a US sample (*N* = 533), we constructed messages to emphasize doctors’ strong recommendation for the COVID-19 bivalent booster. Specifically, we created a reminder that solely referenced doctor endorsements without the ownership language or link (Doctor Recommendation Only arm). By comparing this arm with the holdout (no reminder) arm, we could test the transferability of the findings from Bartoš et al.^27^ to our setting. This is because in Bartoš et al.^27^ people who received information about doctors’ strong endorsement of the COVID-19 vaccine were compared with those who did not receive any reminder or encouragement to get vaccinated. In two additional messages, we combined doctor endorsement with the ownership language and appointment scheduling links (Doctor Recommendations & Ownership w/ Narrow Link and Doctor Recommendations & Ownership w/ Broad Link arms) to examine the additive benefits of highlighting doctors’ recommendations in reminders that otherwise contained only the ownership language and links.

As part of the first RCT, we also compared two strategies for providing appointment scheduling links. The first strategy, akin to Dai et al.^26^, involved providing a link to a specific vaccination venue where individuals could schedule their appointment (the two Narrow Link arms). The second strategy, as adopted by Rabb et al.^32^, involved providing a link to a general website that listed various locations offering the bivalent booster (the two Broad Link arms).

The second RCT (*N* = 159,195; ref. ^41^) examined interventions that we developed based on a survey of COVID-19-related beliefs and that we found effective at changing hypothetical booster intentions in a concurrent online experiment. This RCT compared a basic message (Simple–No Info), which simply told patients they could now get the COVID-19 bivalent booster, with five messages containing additional content aimed at elevating patients’ vaccination intentions. Specifically, based on a survey of 533 California residents we conducted, we crafted three messages to update patients’ beliefs about (1) the differences between the COVID-19 vaccines they had already received and the new booster (Info–Uniqueness), (2) who were eligible for and could benefit from the bivalent booster (Info–Eligibility Clarification), and (3) the severity of COVID-19 symptoms and the effectiveness of the bivalent booster (Info–Severity). In addition, drawing on psychological research on persuasion^42^, we sought to leverage the consistency principle to elevate patients’ intentions to get the bivalent booster. Since patients in our sample had completed the primary COVID-19 vaccine series, we tested the effect of complimenting patients on their completion of the primary series (Consistency) as well as the impact of combining this intervention with information about the uniqueness of the bivalent booster (Consistency & Info–Uniqueness). Critically, while the RCTs were going on, we also assessed the impact of these interventions in a pre-registered experiment on Amazon Mechanical Turk (MTurk) using CloudResearch with 1,774 adults living in the United States who were eligible for the bivalent booster at the time of the study^43^.

The third RCT (*N* = 90,968; ref. ^44^) examined interventions derived from the behavioural literature and forecasted to be effective by experts. These interventions were designed to remind patients that they could get a flu shot alongside the COVID-19 booster shot. Bivalent boosters became available during the flu season in 2022 and public health experts generally recommended getting both the booster and the flu shot. Hence, reminding people that they could receive the two vaccines during the same appointment aimed to reduce the inconvenience associated with obtaining the vaccines separately (for example, the time and cognitive costs associated with scheduling and attending two appointments). In fact, in response to public inquiries about obtaining both vaccines at once, the Centers for Disease Control and Prevention declared in Fall 2022 that it is safe and convenient to do so^45^, and public health officials strongly advocated for receiving both vaccines together as highlighted in major news outlets^46,47^. Further, in the 2022 and 2023 flu seasons, national pharmacies such as Walgreens and CVS Pharmacy prompted customers to receive both vaccines during a single visit, by both sending out text messages and encouraging customers who were booking the appointment for one vaccine to add the other vaccine (Supplementary Notes).

To evaluate this seemingly intuitive strategy, our third RCT compared a simple text message inviting patients to obtain the booster shot (Simple–Enhance Protection) with two messages that additionally reminded patients about the possibility of getting the flu shot and the COVID-19 booster simultaneously. One of the bundling messages encouraged patients to get the COVID-19 booster and tag along the flu vaccine on the same visit (Bundle–Tagging Flu Shot), and the other message encouraged patients to get both the flu shot and COVID-19 booster at the same time (Bundle–Booster & Flu Shot). To confirm that experts shared our intuition that the bundling messages would outperform a simple reminder given prior literature, we recruited 40 attendees at two behavioural science conferences to predict which of the three messages tested in the third RCT would be most effective in promoting booster uptake. These predictions were collected shortly after our trials had concluded but before we had access to the data. We similarly collected predictions from 498 adults living in the United States from Prolific. Also, across two pre-registered online experiments (refs. ^48,49^), we recruited 1,362 adults living in the United States from Prolific—among whom 989 were eligible for the bivalent booster—to evaluate the messages from the third RCT in a between-subjects manner.

## Results

Our analysis includes 314,824 patients who satisfied all the pre-registered inclusion and exclusion criteria described in Methods (for example, not having already received a bivalent booster before the message date). Those patients were, on average, 49.96 years old (standard deviation (s.d.) 17.80), 42.20% were male, 48.76% were white (excluding Hispanic patients) and 14.21% were Hispanic (see Extended Data Table 1 for demographics by condition and balance checks).

We extracted patients’ vaccination records from the California Immunization Registry (CAIR), the most comprehensive database for tracking vaccinations obtained across pharmacies and health clinics in California (including UCLA Health). We complemented CAIR with Epic’ s interoperability platform to capture vaccinations outside California that were documented in patients’ electronic health records. We constructed a pre-registered primary outcome measure to indicate whether patients obtained a COVID-19 bivalent booster within 4 weeks of their assigned message date. We used ordinary least squares (OLS) regressions with heteroskedasticity-robust standard errors to predict booster uptake within 4 weeks while controlling for the pre-registered covariates, including patient gender (male and female, with people whose gender was ‘other’ or unknown to us as the reference group), age, race/ethnicity (Hispanic, White non-Hispanic, Black non-Hispanic, Asian non-Hispanic, and other or mixed races, with people whose race was unknown to us and whose ethnicity was not Hispanic as the reference group), and time when they were assigned to receive the message. Table 2 outlines the key research questions we address using data from the three RCTs, the conditions under comparison for each question, and the corresponding regression results.

**Table 2:** Overview of questions addressed using data from RCTs

Question of interest	RCT(s) analysed	Conditions under comparison	Result location
Impact of reminders	Megastudy-level analysis	All reminders (independently and combined) versus Holdout	Supplementary Table 1 column 1 and column 2
Impact of reminder with ownership language and narrow link	Megastudy-level analysis	Ownership w/ Narrow Link versus Holdout	Supplementary Table 1 column 1
Impact of adding ownership to reminders with links (exploratory)	Megastudy-level analysis	Comparison 1: Four reminders containing ownership language and links versus Nine reminders with links but not ownership Comparison 2: Ownership w/ Narrow Link + Ownership w/ Broad Link versus Simple–No Info + Simple–Enhance Protection	Supplementary Table 1 column 3 and column 4
Impact of adding ownership and link to reminder with doctor recommendation	RCT1	Doctor Recommendation & Ownership w/ Narrow Link + Doctor Recommendation & Ownership w/ Broad Link versus Doctor Recommendation Only	Supplementary Table 2 column 1
Impact of reminder with doctor recommendation	Megastudy-level analysis	Doctor Recommendation Only versus Holdout	Supplementary Table 1 column 1
Impact of adding doctor recommendation to reminders with ownership and link	RCT1	Doctor Recommendation & Ownership w/ Narrow Link + Doctor Recommendation & Ownership w/ Broad Link versus Ownership w/ Narrow Link + Ownership w/ Broad Link	Supplementary Table 2 column 1
Impact of directing people to a specific vaccination venue (versus a general website with numerous venues)	RCT1	Ownership w/ Narrow Link + Doctor Recommendation & Ownership w/ Narrow Link versus Ownership w/ Broad Link + Doctor Recommendation & Ownership w/ Broad Link	Supplementary Table 2 column 2
Impact of adding booster-related information to simple reminder	RCT2	Info–Uniqueness + Info–Eligibility Clarification + Info–Severity versus Simple–No Info	Supplementary Table 4 column 2
Impact of adding consistency language to simple reminder	RCT2	Comparison 1: Consistency versus Simple–No info Comparison 2: Consistency & Info–Uniqueness versus Simple–No info	Supplementary Table 4 column 2
Impact of adding bundling language to simple reminder	RCT3	Bundle–Tagging Flu Shot + Bundle–Booster & Flu Shot versus Simple–Enhance Protection	Supplementary Table 6 column 2

This table summarizes questions that we attempt to address using data from the three RCTs, the RCT(s) analysed to answer each question, the conditions under comparison in each analysis, and where to locate the corresponding regression results.

### Megastudy and RCT1: interventions with prior field support

The megastudy design and the first RCT allowed us to investigate interventions built upon field work that emerged during the early stages of COVID-19 vaccine rollout. We provide evidence on the impact on vaccinations of the following: (1) sending reminders, as compared with the no-reminder arm; (2) leveraging psychological ownership through the ‘claim your dose’ language; (3) referencing doctors’ endorsement of vaccines; and (4) including narrow as opposed to broad links to facilitate appointment scheduling.

**Fig. 2:**
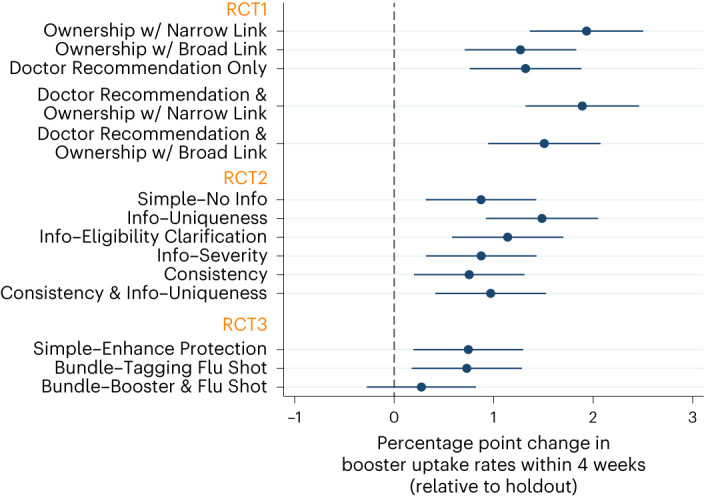
Regression-estimated increase in booster uptake induced by each message in the RCTs, relative to the holdout. This figure shows the regression-estimated increase in bivalent booster uptake rates within 4 weeks of the assigned message date, induced by receiving a given text message (versus holdout) for the first RCT (top), second RCT (middle) and third RCT (bottom). The data are presented as average treatment effects with 95% CIs, derived from an OLS model (see Supplementary Table 1 column 1 for the full statistics, and see ref. ^50^ for adjusted *P* values after correcting for 14 comparisons of individual messages with the holdout). The bivalent booster uptake rate was 12.39% in the holdout condition. The full sample refers to 314,824 patients in the analysis sample, and Fig. 1 shows the number of patients in each condition.

First, to estimate the effect of reminders on booster uptake, we leverage our megastudy design and analyse data from all three RCTs. Figure 2 reports the regression-estimated change in booster uptake rates induced by the 14 text-based interventions (versus the holdout condition), which corresponds to column 1 in Supplementary Table 1. Relative to the holdout condition where 12.39% of patients received the bivalent booster within 4 weeks, all but one reminder significantly increased booster uptake by 0.73 percentage points to 1.93 percentage points (*t*(314,769) ranging from 2.59 to 6.65; *P* values ranged from <0.001 to 0.01). The exception is the Bundle–Booster & Flu shot message (*B* = 0.0027, *t*(314,769) = 0.98, *P* = 0.33, 95% confidence interval (CI) −0.0027 to 0.0082). The findings hold after we correct for the 14 comparisons between individual messages and the holdout condition, calculate the Romano–Wolf stepdown-adjusted *P* values, and control the family-wise error rate at the 0.05 level (see ref. ^50^ for Romano–Wolf stepdown-adjusted *P* values). The average effect of all reminders on booster uptake within 4 weeks was 1.13 percentage points (*t*(314,782) = 7.33, *P* < 0.001, 95% CI 0.0083 to 0.0143; Supplementary Table 1 column 2).

Second, we examine interventions that encouraged vaccinations using the ‘claim your dose’ language in several ways. Relative to the holdout condition, the Ownership w/ Narrow Link condition increased the booster uptake rate by 1.93 percentage points (*t*(314,769) = 6.65, *P* < 0.001, 95% CI 0.0136 to 0.0250; Fig. 2, top). Whereas the design of the first RCT alone does not allow us to examine whether adding the ‘claim your dose’ language to a reminder increases vaccinations more than a basic reminder, we can provide some insights via exploratory analyses that leverage the megastudy design. Specifically, we can compare messages that included the ownership language to messages that did not. Among 13 messages with links to vaccination venues, the messages containing the ‘claim your dose’ language led to significantly higher booster uptake by 0.78 percentage points, compared with other reminders lacking such ownership language (*t*(240,733) = 5.15, *P* < 0.001, 95% CI 0.0048 to 0.0107; Supplementary Table 1 column 3). Also, the two text messages that contained only the ‘claim your dose’ language without doctor recommendations (that is, Ownership w/ Narrow Link and Ownership w/ Broad Link) increased booster uptake by 0.79 percentage points, relative to the simple reminders in the second and third RCTs (that is, Simple–No Info and Simple–Enhance Protection; *t*(74,193) = 3.17, *P* = 0.002, 95% CI 0.0030 to 0.0128; Supplementary Table 1 column 4). Finally, we perform a pre-registered comparison between the Doctor Recommendation & Ownership w/ Narrow Link arm, the Doctor Recommendation & Ownership w/ Broad Link arm, and the Doctor Recommendation Only arm in the first RCT. This comparison does not show statistically significant evidence for the benefits of adding the ownership language to a reminder with doctor endorsement information (*B* = 0.0037, *t*(92,464) = 1.19, *P* = 0.23, 95% CI −0.0024 to 0.0097; Supplementary Table 2 column 1).

Third, we investigate the impact of interventions that highlighted doctors’ endorsement of the vaccine. Since prior field work^27^ found that information about doctors’ endorsement of the COVID-19 vaccine increased vaccinations relative to a no-message control, we begin with a comparison between the holdout condition and the reminder that only included language about doctor endorsement without a link to appointment scheduling (Doctor Recommendation Only condition). We find that this reminder led to a higher booster uptake rate by 1.32 percentage points, relative to the holdout condition (*t*(314,769) = 4.62, *P* < 0.001, 95% CI 0.0076 to 0.0188; Fig. 2, top), in line with the key finding in Bartoš et al.^27^. Unfortunately, we cannot discern the specific impact of doctor endorsement in isolation from the impact of receiving a reminder, since our RCTs did not include a reminder that lacked both appointment links and doctor endorsement information. We further compare the two conditions containing both doctor endorsement and ownership language (that is, Doctor Recommendation & Ownership w/ Narrow Link and Doctor Recommendation & Ownership w/ Broad Link) with the two conditions containing only ownership language (that is, Ownership with Narrow Link and Ownership with Broad Link). There, we find no detectable benefits of adding doctor recommendations to reminders that already included ownership language and appointment scheduling links (*B* = 0.0009, *t*(92,464) = 0.37, *P* = 0.72, 95% CI −0.0040 to 0.0059; Supplementary Table 2 column 1).

Finally, using data from the first RCT, we conduct a pre-registered comparison of two approaches to incorporating links to appointment scheduling websites, which were either used by Dai et al.^26^ or Rabb et al.^32^. We find that the two messages directing people to a specific vaccination venue (Ownership w/ Narrow Link and Doctor Recommendations & Ownership w/ Narrow Link) led to a 0.51 percentage point increase in booster uptake compared with the two messages directing people to various vaccine venues on a general website (Ownership w/ Broad Link and Doctor Recommendation & Ownership w/ Broad Link; *t*(73,991) = 2.03, *P* = 0.043, 95% CI 0.0002 to 0.0101; Supplementary Table 2 column 2). While the difference is small in magnitude and hovers near the 5% significance threshold, it provides some suggestive evidence that removing flexibility and guiding individuals to a specific location may enhance convenience and reduce the cognitive and time cost of choosing a vaccine venue. This result may contribute to explain the discrepant findings between Dai et al.^26^—which documented a positive impact of text reminders containing a narrow link—and Rabb et al.^32^—which precisely estimated a null effect of reminders containing a broad link, among other potential explanations such as differences in the targeted population’s vaccine hesitance^51^. Supplementary Notes presents information about the percentage of patients receiving their boosters at the specific vaccination venue that our narrow link directed them to (CVS Pharmacy).

### RCT2: interventions with support from a hypothetical study

**Fig. 3:**
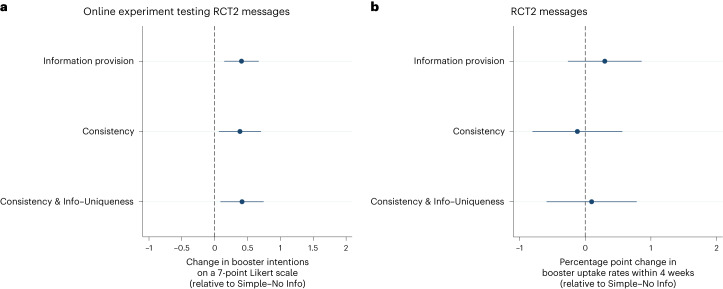
Regression-estimated difference between the basic reminder and other messages tested in the online experiment versus the second RCT. **a**,**b**, The regression-estimated differences in bivalent booster uptake intentions (**a**) and bivalent booster uptake rates (**b**) between the Simple–No Info message and other messages tested in the second RCT. **a** comes from data on 1,774 participants in an online experiment who reported intentions to receive the bivalent booster on a 7-point Likert scale. **b** comes from data on 111,126 UCLA Health patients in one of the six message conditions in the second RCT, for whom we assessed whether they actually received the bivalent booster within 4 weeks of the assigned message date. Both panels compare the three information provision messages (Info–Uniqueness, Info–Eligibility Clarification and Info–Severity combined), Consistency message, and Consistency & Info–Uniqueness message with the Simple–No Info message. The data are presented as average treatment effects with 95% CIs, derived from OLS models (see Supplementary Table 3 column 5 and Supplementary Table 4 column 2 for full statistics, and see ref. ^50^ for adjusted *P* values after correcting for three comparisons in **a**).

In an MTurk experiment (*N* = 1,774) we evaluated whether the strategies used in the second RCT seem persuasive to people eligible for the bivalent booster and could enhance their intentions to get the bivalent booster. There, compared with the Simple–No Info message, the three information interventions combined and the consistency-based interventions were rated as more persuasive (*B* ranged from 0.326 to 0.913 on a 1–7 Likert scale, *t*(1,762) ranged from 2.46 to 6.75, *P* values ranged from <0.001 to 0.014). They also elevated intentions to obtain the booster, relative to the Simple–No Info message (*B* ranged from 0.385 to 0.417 on a 1–7 Likert scale, *t*(1,762) ranged from 2.35 to 3.06, *P* values ranged from 0.002 to 0.019; Fig. 3a and Supplementary Table 3). These findings survive multiple hypothesis testing (see ref. ^50^ for Romano–Wolf stepdown-adjusted *P* values).

While these results may suggest that addressing important misconceptions about bivalent boosters and leveraging consistency could be promising strategies for motivating booster uptake, the results from the field painted a less positive picture (Fig. 3b and Supplementary Table 4). Turning to the second RCT, we find that compared to the Simple–No Info message, neither providing information about the bivalent booster nor leveraging the consistency principle further increased booster uptake (*B* ranged from −0.0012 to 0.0030, *t*(111,082) ranged from −0.34 to 1.04, *P* values ranged from 0.30 to 0.78). Altogether, while information interventions aimed at changing beliefs about boosters and interventions leveraging the consistency principle have demonstrated potential in influencing intentions to obtain boosters hypothetically, they do not yield meaningful benefits on actual booster uptake in the field.

### RCT3: interventions favoured by third-party experts and laypeople

In predicting the most effective message from the third RCT, experts at behavioural science conferences (*N* = 40) and laypeople from Prolific (*N* = 498) selected messages containing the bundle interventions (Bundle–Tagging Flu Shot and Bundle–Booster & Flu Shot messages) at a higher rate than the chance level of 66.6% (85.00%, *z* = 2.47, *P* = 0.014 for experts, 92.97%, *z* = 12.48, *P* < 0.001 for laypeople). In particular, they selected the Bundle–Booster & Flu Shot message at the highest rate, surpassing the chance level of 33.3% (60.00%, *z* = 3.58, *P* < 0.001 for experts, 70.48%, *z* = 17.61, *P* < 0.001 for laypeople). Across two online experiments where laypeople were presented with one of the three messages (total *N* = 989), we observe additional evidence in favour of the bundling strategy. Specifically, compared with the simple reminder, the two bundling messages combined increased the perceived convenience of receiving the flu shot along with the booster (*B* = 1.749, *t*(850) = 13.00, *P* < 0.001, 95% CI 1.485 to 2.012) and were rated as more persuasive (*B* = 0.427, *t*(979) = 4.08, *P* < 0.001, 95% CI 0.222 to 0.633), though they only directionally improved booster uptake intentions (*B* = 0.096, *t*(979) = 0.75, *P* = 0.45, 95% CI −0.154 to 0.346; all on the 1–7 Likert scale; Supplementary Table 5).

However, the field data from the third RCT do not align with the predictions of experts and laypeople as well as the results of the online experiments. Specifically, our third RCT found that the two bundling messages combined did not outperform the Simple–Enhance Protection reminder (*B* = −0.0025, *t*(55,574) = −0.82, *P* = 0.41, 95% CI −0.0083 to 0.0034; Supplementary Table 6), suggesting that this strategy did not meaningfully impact behaviour in our field context.

## Discussion

Applied behavioural science has undergone tremendous growth in recent decades. However, critiques have emerged regarding its ability to have meaningful impacts at scale, leading prominent figures from academia and beyond to argue that further development is necessary for behavioural science to effectively influence real-world practices^9,20^. Effectively translating behavioural science research to practices and identifying behavioural interventions that can change consequential behaviours across settings requires concerted efforts from scientists, practitioners and funders. Such efforts can aid the development of a nuanced understanding of whether and when behavioural science knowledge is applicable and transferrable across contexts^12^.

Developing such an understanding requires contributions from multiple perspectives. For instance, minimizing flawed evidence and adhering to best methodological practices^52,53^ can bolster confidence in the reliability of empirical findings. Further, investigating moderators of intervention effectiveness can help identify actionable insights that can be translated across contexts^20,54^. Ongoing research has recognized that nuanced differences in measurement^51^ and population^33,51,55–58^, as well as contextual moderators and individuals’ sense-making processes, may contribute to differences in intervention effect sizes or even cause interventions that seem promising in prior work to backfire unexpectedly^59,60^. This paper aims to extend these endeavours by assessing the transferability of evidence from various sources to real-world scenarios.

Our findings highlight the importance of accumulating knowledge about intervention impact in the real world, which could help practitioners save critical resources when attempting to influence consequential behaviours. In our setting, adding to reminders behavioural interventions that showed promising results online (RCT2) or were expected to work by behavioural science experts and laypeople (RCT3) showed little efficacy in the field. While hypothetical surveys and self-reports are undoubtedly valuable for providing foundational evidence on the mechanisms of human behaviour, our findings suggest that they may not always translate to complex real-world situations where various factors can affect behaviour. We should note that our findings are specific to the context of COVID-19 vaccinations in a particular setting, which could limit their generalizability. Nevertheless, our results suggest that conducting field tests of promising interventions beyond self-reports and outside of highly controlled studies, through partnerships between researchers, practitioners and funders, can be beneficial.

Our research also provides one data point on the robustness of interventions identified as effective in prior field evaluations: sending reminders, leveraging psychological ownership to make individuals feel the vaccine is theirs, and providing information about doctors’ endorsement of the vaccine. Replicating past work on promoting initial COVID-19 vaccinations in similar or different populations^26,27,61^, we find that, relative to the holdout condition, receiving a reminder on average significantly increased booster uptake by 1.13 percentage points (or about 9%); the reminder containing the ownership language and a link to a specific vaccine venue, which resembled the best-performing reminder in the first RCT in Dai et al.^26^, and the reminder referencing doctors’ endorsement, which was modelled after Bartoš et al.^27^, increased vaccinations by 1.93 percentage points (or about 16%) and 1.32 percentage points (or about 11%), respectively.

We further find that the ownership language increased uptake by approximately 0.8 percentage points (or about 6%) relative to the messages without this language, consistent with Dai et al.^26^. One limit of our design is that it did not allow us to cleanly test whether adding information about doctors’ endorsement to basic reminders would increase booster uptake, which warrants future research. However, our design did allow us to observe that emphasizing doctors’ endorsement appeared to be a substitute for inducing psychological ownership and directing people to vaccination venues, as combining these interventions did not further yield detectable benefits beyond implementing either of them individually. Future research could investigate how to achieve synergies between interventions that independently seem to work.

While the effect sizes of text reminders and ownership framing in our RCTs are modest, they are noteworthy for two reasons. First, our participants had been repeatedly exposed to COVID-19 vaccination messages. On top of the behaviourally informed reminders many patients in our sample received from UCLA Health to encourage first-dose vaccinations^26^, patients living in the Los Angeles area (approximately 80% of the sample) may have received text messages about COVID-19 vaccinations from the City of Los Angeles in Spring and Summer 2021. Some of these messages leveraged psychological ownership (Supplementary Notes). Additionally, during our observation period, patients might have received text messages encouraging COVID-19 booster uptake from pharmacies like Walgreens and CVS Pharmacy (Supplementary Notes), which could have further hindered the impact of our reminders. Despite this messaging saturation, reminders and ownership framing interventions showed a similar magnitude of impact to what was observed for similar messages encouraging uptake of the first dose of the COVID-19 primary vaccine series^51^ (Supplementary Notes). Second, the modest impact in our work is documented on booster vaccinations anywhere over 4 weeks, an outcome measure that is probably harder to move than indicators of interest such as web traffic or clicking rates^62^. These findings provide some evidence that text reminders and ownership framing can remain effective even when deployed multiple times within the same population, which may have broader implications for motivating repeated behaviour change. However, further research is needed to understand how to determine the optimal spacing of such interventions to maintain their impact.

It is important to note that our interventions may not work universally^32,33^. Prior work suggests that the impact of reminders may vary across subpopulations facing different barriers to adopting a behaviour. For example, reminders may not always work when deployed among low-income populations^34^. We took advantage of our large sample to provide some insight into this question by comparing the average effect of reminders between subpopulations with relatively low socio-economic status and subpopulations with relatively high socio-economic status. We made this distinction as proxied by their social vulnerability index and the income and education levels in their neighbourhoods. While our exploratory analyses did not find detectable differences in the impact of reminders between these subpopulations (Supplementary Table 7), further research is needed to understand the conditions under which these and other behaviourally informed light-touch interventions may lose their potency.

The field of behavioural science has undergone major changes in the past decades. Growing concerns about the replicability and reliability of scientific findings have sparked a much-needed conversation about the importance of scientific rigour^63^. In response, researchers have recognized the critical role of reproducibility in building a solid foundation of trustworthy evidence. While a large and growing number of studies have focused on replicating laboratory findings across the social sciences^64–66^, replications in real-world contexts have been infrequent. Our research takes a stride in this direction by assessing the transferability of insights gained in one field context to another, and from hypothetical and prediction surveys to field settings. From a theoretical perspective, identifying which findings are robust and documenting their boundary conditions can help build stronger theories of behaviour change. From a practical standpoint, these efforts are essential for helping academics, practitioners, consultants and other decision-makers keen on leveraging behavioural science to make more informed assessments about the transferability of behavioural insights and identify intervention that are more likely to work in a given organizational or policy context.

## Methods

This research was deemed to comply with all relevant ethical regulations. The Institutional Review Board at the UCLA approved the protocols of the RCTs (reference number 21-000268) and determined that a waiver of informed consent was appropriate. The online experiments and the vaccination intention survey were conducted under approval of the Institutional Review Board at Carnegie Mellon University (reference number STUDY2020_00000347), and informed consent was obtained from online study participants as part of the enrolment process. The expert prediction survey was deemed as non-human subject research by the Institutional Review Board at UCLA.

The three RCTs were officially pre-registered at clinicaltrials.gov on 19 October 2022 (RCT1, ref. ^40^; RCT2, ref. ^41^; RCT3, ref. ^44^), though the investigators submitted the study record a few days in advance. The online experiment accompanying RCT2 was pre-registered on 25 October 2022 (ref. ^43^). The online experiments accompanying RCT3 were pre-registered on 4 November 2022 (ref. ^48^) and 14 November 2022 (ref. ^49^). All of our (experimental, prediction or correlational) surveys were executed via Qualtrics, and their materials are available at ref. ^50^.

For the RCTs, enrolment was conducted by the UCLA Health Office of Population Health and Accountable Care, random assignment to interventions was performed by UCLA Health statisticians blind to the hypotheses and interventions using a computerized random number generator, and messages were sent by Artera (WELL Health)—UCLA Health’s text messaging vendor. The investigators were blind to condition assignment during the experimental period. For online experiments, the randomization was implemented at the individual level with Qualtrics randomizers. The experiments were double blind in the sense that participants were not informed of their own treatment assignment, and the experiment administration was automated. Analysis was not performed blind to the conditions of the field and online experiments.

### Setting, enrolment and design of the RCTs

We conducted three parallel RCTs in partnership with UCLA Health, a large health system in California. On 14 September 2022, UCLA Health sent a mass email to patients informing them of the authorization of COVID-19 bivalent boosters, providing a timeline for when UCLA Health would begin offering bivalent booster appointments, and advising patients to get the bivalent booster from a local pharmacy if they would like to get it sooner.

Our enrolment criteria include all UCLA Health primary care or specialty attributed patients who: (1) completed the COVID-19 primary vaccine series by 10 October 2022, (2) had not received any COVID-19 dose within 2 months before that date, (3) were at least 18 years old and (4) had a phone number on file that had not previously been opted out of UCLA Health text messaging. This process identified 386,615 eligible patients.

Since UCLA Health wanted to limit the number of messages sent out at any given point in time, we sent text messages across three time slots (9:00, 12:00 and 16:00) on 11 workdays (from 18 October 2022 to 1 November 2022). Our randomization process ensured that the chance of being randomly assigned to any given message condition was the same across the 14 messages conditions, and that the chance of being randomly assigned to not getting a reminder was three times the chance of being randomly assigned to one message condition. We oversampled patients who did not get a reminder because we had three parallel RCTs. Specifically, we first randomly assigned all 386,615 patients to the first, second and third RCT at a 6:7:4 ratio (with 6, 7 and 4 corresponding to the number of conditions, including a holdout condition, in each RCT). Then within the first RCT, 136,452 patients were randomly assigned to one of six conditions (five message conditions and one no-reminder holdout condition) with an equal probability; within the second RCT, 159,195 patients were randomly assigned to one of seven conditions (six message conditions and one no-reminder holdout condition) with an equal probability; and within the third RCT, 90,968 patients were randomly assigned to one of four conditions (three message conditions and one no-reminder holdout condition) with an equal probability. Within each condition, we randomly assigned 680 patients to each of the three time slots (9:00, 12:00 and 16:00) on 18 October, 19 October, 20 October, 21 October, 27 October, 28 October, 31 October and 1 November 2023, and randomly assigned the remaining patients at an equal ratio to each of the three time slots on 24 October, 25 October and 26 October 2023 (with each slot getting 713 or 714 patients). Though we randomized some patients into a holdout condition within each RCT, our analyses follow the megastudy approach to combine all three no-reminder holdout conditions into one big holdout condition whenever we compare patients who received a reminder with patients who did not receive a reminder. Figure 1 and Supplementary Table 8 present the number of patients assigned to each day, each time slot and each condition.

During the first three days of our RCTs, each message (except for the Doctor Recommendation Only message) included two links: one directed people to make an appointment at UCLA Health; the other directed people to book an appointment somewhere else—either with a link to CVS Pharmacy or a link to a general website with numerous vaccine venues (www.vaccines.gov). Details about the links are provided in Supplementary Methods.

After we launched the RCTs, we learned that appointment availability at UCLA Health was more limited than UCLA Health had expected due to staff shortage. Thus, starting from the fourth day of the RCTs (that is, 21 October 2023), we removed the link to UCLA Health from all messages with links, acknowledged the limited supply of appointments at UCLA Health in the messages, and encouraged patients to get the bivalent booster somewhere else. Notably, the essence of the messages (for example, the psychological principles leveraged and the information provided about the bivalent boosters) remained the same, and the change was applied to all messages. We updated our pre-registrations as soon as the changes occurred. Table 1 in Main presents the messages used from the fourth day of our RCTs onwards (since the majority of patients in our sample were enrolled in our RCTs from the fourth day onwards). The exact wording of text messages used in the first three days can be found at ref. ^50^.

Due to a technical error, our text messaging vendor sent a small percentage of patients (0.34%) messages from two conditions. Also, a small number of patients (1.67%) did not receive their assigned message because of technical errors, invalid phone numbers or patients opting out of receiving messages from the short code that the vendor sent messages from. We report the intent-to-treat results in Main using each patient’s randomly assigned condition, regardless of whether they actually got the message. All of the results reported in Main are robust if we remove patients who did not receive their assigned message and if we further remove patients who received two messages by mistake, as shown at ref. ^50^.

### Exclusion criteria and balance checks for the RCTs

At the analysis stage, we applied our pre-registered exclusion criteria to the 386,615 enrolled patients. First, our analyses exclude patients who received any dose of the COVID-19 vaccine within the 2 months (or precisely 60 days) before their assigned message date, because those patients were not eligible to receive the bivalent booster at the time of getting our message. Though we already tried our best to take into account whether patients received a dose within 2 months before 10 October 2022 (that is, when we selected the pool of patients to enroll), vaccination records get updated over time, and some patients may get a dose between 10 October 2022 and their assigned message date.

Further, we exclude patients who, as far as UCLA Health could track, received the COVID-19 bivalent booster before the assigned message date, or had died before the study. We also pre-registered that we would exclude patients who scheduled a booster appointment at UCLA Health before the assigned message time. However, we ended up not using the data about appointments for two reasons. One is that the staff shortage at UCLA Health meant that only a limited number of patients were able to schedule bivalent booster appointments there. The other reason is that we learned after the RCTs ended that some patients were able to get a bivalent booster at a doctor visit during our experiment period without making a bivalent appointment, which means that, before their assigned message time, some patients may have already planned to get the booster at their upcoming normal doctor appointments but we could not tell it from the bivalent booster appointment data.

Importantly, the proportion of patients excluded from the analysis stage did not statistically significantly differ across conditions, as expected (Extended Data Table 1). Figure 1 shows the number of participants who were included in the analysis sample in each condition (in addition to the number of patients assigned to each condition).

To test whether our study arms were well balanced, we predicted balance variables—including an indicator for whether patients were retained in the analysis, patient age (in years), and an indicator for whether a patient was male—as a function of experimental conditions using OLS regressions with HC3 heteroskedasticity-robust standard errors. *F*-tests were then conducted for the *β* coefficients from the regressions to compare the overall significance across relevant conditions. To summarize across all categories of race/ethnicity, we analysed the categorical variable of race/ethnicity using a chi-squared test. Extended Data Table 1 shows that the conditions were balanced on the rate of being retained in the analysis, gender, age and race/ethnicity within each RCT as well as for the 15 conditions across three RCTs, except that race/ethnicity was slightly unbalanced within the second RCT (${{\mathscr{X}}}^{2}(25)$ = 38.31, *P* = 0.043). As explained later, our pre-registered regression specifications control for race/ethnicity.

### Statistical analysis of the RCTs

Our pre-registered primary outcome measure is Booster Uptake, a binary measure of whether patients obtained a COVID-19 bivalent booster within 4 weeks of their assigned message date. To capture COVID-19 vaccinations as comprehensively as possible, we primarily rely on administrative records from the CAIR, which we complement with additional vaccination records from organizations that participate in Epic’s healthcare information exchange.

Following the pre-registrations, we report OLS regressions that predict Booster Uptake. All regressions, unless otherwise explained, include the pre-registered controls mentioned in Main. The binary outcome measure of Booster Uptake violated both normality and homoskedasticity assumptions, but our pre-registered analysis involves OLS regressions because OLS regressions are recommended for estimating treatment effects on binary outcomes in experiments^67^, and all of our regressions use heteroskedasticity-robust standard errors. All results about the RCTs reported in Main are robust to using logistic regressions (as shown at ref. ^50^). As an additional robustness check requested by the reviewer team, we explore whether patients received the bivalent booster within 8 weeks of the assigned message date and obtain qualitatively similar though sometimes less precise results as what are reported in Main (see ref. ^50^).

All analyses reported in Main and Supplementary Information about the RCTs and companion surveys use two-tailed tests and are performed in Stata 14. In Supplementary Methods, we explain the regression specifications used to answer each question of interest. Supplementary Tables 1, 2, 4 and 6 report regression results.

### Online experiment examining messages tested in RCT2

During 25–28 October 2022, while our RCTs were ongoing, we conducted a pre-registered online experiment to examine the interventions implemented in our second RCT. We sought to test how these interventions affect people’s intentions to get the booster and their perceived persuasiveness of these messages.

Using CloudResearch, we recruited adults from MTurk who were living in the United States, were eligible for the COVID-19 bivalent booster, and had not yet received it (see Supplementary Methods for details about these selection criteria). We asked participants to imagine that their healthcare provider texted them about the bivalent COVID-19 boosters. Participants were randomly assigned to read one of the six messages tested in our second RCT with minor modifications to suit the context. All messages explained to patients that ‘Our clinics have limited booster appointments available’, and encouraged patients to book an appointment at a pharmacy nearby, which mimicked the text messages used in our second RCT from the fourth day on.

After reviewing the assigned message, participants rated how persuasive they thought the message was (from 1 (Not at all persuasive) to 7 (Very persuasive)) and how likely they would be to get the bivalent COVID-19 booster (from 1 (Not at all likely) to 7 (Very likely)). Persuasiveness and Booster Intentions were our pre-registered dependent variables, and their order was randomized.

Next, participants responded to a series of questions assessing their beliefs about the bivalent COVID-19 booster (see ref. ^50^ for the full survey). In the end, participants reported demographics and their COVID-19 vaccination history, among other background information.

A total of 1,774 participants met our aforementioned selection criteria, responded to our pre-registered outcome measures and were thus included in our analysis. They were an average of 42.06 years old (s.d. 12.92), 47.69% were male, 73.56% were white (excluding Hispanic participants) and 4.28% were Hispanic. We aimed to obtain 300 participants per message, to have 80% statistical power to detect differences of a small magnitude (Cohen’s *d* around 0.25) between the Simple–No Info message and each of the other treatment messages.

Results reported in Main come from OLS regressions with HC3 heteroskedasticity-robust standard errors, which controlled for pre-registered covariates of gender (male and female, with people whose gender was ‘other’ or unknown to us as the reference group), age, race/ethnicity (Hispanic, white non-Hispanic, Black non-Hispanic, and Asian non-Hispanic, with people whose race was other or mixed or unknown to us and whose ethnicity was not Hispanic as the reference group), and an indicator for missing demographics. For participants with missing demographics information, age was set to be at the mean level for the regression analyses. In Supplementary Methods, we explain the OLS regression specifications used to answer each question of interest, and any deviation from the pre-registration. Supplementary Table 3 reports regression results.

Our data about Perceived Persuasiveness and Booster Intentions violate the normality assumption. The reported results about the differences between the Simple–No Info message and other treatment messages (that is, the three information provision messages combined, the Consistency message, and the Consistency & Info–Uniqueness message) in Perceived Persuasiveness and Booster Intentions are robust when we use non-parametric Mann–Whitney *U* tests.

### Prediction survey with experts

We invited attendees of two conferences, the 2022 Annual Behavioral Science and Health Symposium (10 November 2022) and the 2022 Society of Judgment and Decision Making annual meeting (11–13 November 2022), to participate in a brief prediction survey. Respondents were presented with a brief background about the third RCT at UCLA Health and asked to select which one of the three text messages tested in the third RCT would lead the largest number of patients to receive the COVID-19 bivalent booster. A total of 40 conference attendees responded to our survey during the two aforementioned conferences, comprising 47.5% faculty members and 45% post-docs, PhD students or other academic positions.

### Online studies examining messages tested in RCT3

In November 2022 (4, 7, 14, and 15 November 2022), we conducted two pre-registered online experiments to examine the vaccine bundling interventions implemented in our third RCT. We recruited adults living in the United States who were eligible for the COVID-19 bivalent booster but had not yet received it. We used Prolific’s screening database to identify participants in the United States who had received at least one dose of the COVID-19 vaccine and were fluent in English. At the beginning of the survey, we asked participants whether they had completed the COVID-19 primary vaccine series, when they received their last dose of the COVID-19 vaccine, and whether they had already received the flu shot for the 2022–2023 flu season.

We asked participants to imagine that their healthcare provider sent them a text message about the bivalent COVID-19 booster. Participants were randomly assigned to read one of the three messages tested in our third RCT, with minor modifications to fit the context (for example, replacing ‘UCLA Health’ with ‘Our clinics’). Participants then rated how persuasive they thought the message was (from 1 (Not at all persuasive) to 7 (Very persuasive); Perceived Persuasiveness), how likely they would be to get the bivalent COVID-19 booster (from 1 (Not at all likely) to 7 (Very likely); Booster Intentions), and the extent to which the message would make them feel that it was convenient to get the flu shot at the same time as the bivalent COVID-19 booster (from 1 (Not at all) to 7 (Extremely); Perceived Convenience). These variables were pre-registered primary outcome measures in at least one of the online experiments. Participants also responded to a few additional questions about the bivalent booster or flu shot (see ref. ^50^ for the full surveys).

In the first experiment, participants were additionally presented with a brief background about the third RCT at UCLA Health and asked to select which one of the three text messages tested there would lead the largest number of patients to receive the COVID-19 bivalent booster.

At the end of both experiments, participants reported their demographics and COVID-19 vaccination history, among other background information.

For between-subjects analyses that compared participants who were assigned to read one message, our pre-registered plan for both experiments was to focus on participants who had completed the COVID-19 primary vaccine series at the time of our study and did not obtain any vaccine dose since September 2022 to the time of our study. Across the two experiments, a total of 989 participants met these criteria and responded to Perceived Persuasiveness (the pre-registered outcome measure in both experiments). These participants had an average age of 34.78 years old (s.d. 12.80), 48.03% were male, 68.15% were white (excluding Hispanic participants) and 6.27% were Hispanic.

We report analyses that combine the two online experiments. Results reported in Main come from OLS regressions with HC3 heteroskedasticity-robust standard errors, which controlled for pre-registered covariates of gender (male and female, with people whose gender was ‘other’ or unknown to us as the reference group), age, race/ethnicity (Hispanic, white non-Hispanic, Black non-Hispanic, and Asian non-Hispanic, with people whose race was other or mixed or unknown to us and whose ethnicity was not Hispanic as the reference group) and an indicator for missing demographics. For participants who had missing demographics or who clearly entered impossible values as their age (‘4’ and ‘1,981’), age was set to be at the mean level for the regression analyses. In Supplementary Methods, we explain the OLS regression specifications used to answer each question of interest and deviations from the pre-registration in the first experiment. Supplementary Table 5 reports regression results.

Our data about Perceived Persuasiveness, Booster Intentions and Perceived Convenience violate the normality assumption. The reported results about the differences between the Simple–Enhance Protection message and the vaccine bundling messages in these outcome measures are robust when we use non-parametric Mann–Whitney *U* tests.

When analysing the prediction made by laypeople who were presented with all three messages, we focus on 498 Prolific respondents who made a prediction (regardless of whether they had already gotten the bivalent booster), among whom the average age was 37.13 (s.d. 14.21), with 45.58% identifying as male, 72.49% identifying as white (excluding Hispanic participants) and 4.42% identifying as Hispanic. Supplementary Methods presents the results about predictions made by the subset of 363 respondents who had completed the COVID-19 primary vaccine series at the time of our study and did not obtain any vaccine dose in September, October or November 2022.

### Online survey of beliefs associated with vaccination intentions

On 16 September 2022, we conducted an online survey to investigate the factors that predict the general public’s intentions to receive the COVID-19 bivalent booster. We recruited 533 adults from Prolific (*n* = 349) and MTurk via CloudResearch (*n* = 184) who had completed the COVID-19 primary vaccine series, lived in California (according to Prolific’s screening system and CloudResearch’s MTurk toolkit), passed an attention check (for MTurk only), and finished the survey. They were, on average, 37.57 years old (s.d. 13.46), 56.29% were male, 48.78% were white (excluding Hispanic participants), 13.13% were Hispanic and 97.19% were living in California at the time of the study. To select participants on Prolific based on their vaccination status, we used Prolific’s screening system to identify people who had received at least one dose of the COVID-19 vaccine. We confirmed that all participants in our final sample had completed the COVID-19 primary vaccine series based on their self-reports. For MTurk, only participants who reported having completed the primary vaccine series were allowed to take the survey, and participants were unaware of our selection criterion when they provided their vaccination status.

At the beginning of the survey, we informed participants that the Food and Drug Administration had authorized the use of the new bivalent COVID-19 boosters developed by Pfizer and Moderna. We elicited intentions to get the bivalent booster, and asked a series of questions about their beliefs regarding the coronavirus and the bivalent booster. The measures that were particularly important to shaping the design of our text messages assessed participants’ beliefs about their eligibility for the bivalent booster, infection likelihood with and without the bivalent booster, Long COVID likelihood with and without the bivalent booster, infection severity with and without the bivalent booster, the comparative efficacy between the bivalent booster and the original COVID-19 booster, confusion about public health guidelines, and doctor recommendation. We compared answers to these questions among people who planned to get the booster (including those who already got it and those who had not received it), those who were uncertain, versus those who did not plan to get the booster. Supplementary Methods and Supplementary Table 9 describe these variables and results, and the full survey can be found at ref. ^50^.

Based on the results, we developed text messages to address different factors associated with booster uptake intentions. Specifically, our messages (1) emphasized the enhanced efficacy of bivalent boosters over original vaccines in fighting against the dominant Omicron variants (Info–Uniqueness), (2) clarified potential confusion about who were eligible for and could benefit from the bivalent boosters (Info–Eligibility Clarification), (3) highlighted the chance of developing Long COVID and severe COVID-19 symptoms as well as the effectiveness of bivalent boosters in reducing the risk (Info–Severity), and (4) communicated doctors’ strong recommendations (Doctor Recommendation Only, Doctor Recommendation & Ownership w/ Narrow Link, Doctor Recommendation & Ownership w/ Broad Link). In addition, all messages either clearly informed patients that they were eligible for the bivalent booster or implied so.

### Reporting summary

Further information on research design is available in the Nature Portfolio Reporting Summary linked to this article.

### Supplementary information

Supplementary Information Supplementary Tables 1–10, Methods and Notes.

Reporting Summary

## Figures and Tables

**Extended Data Table 1:**
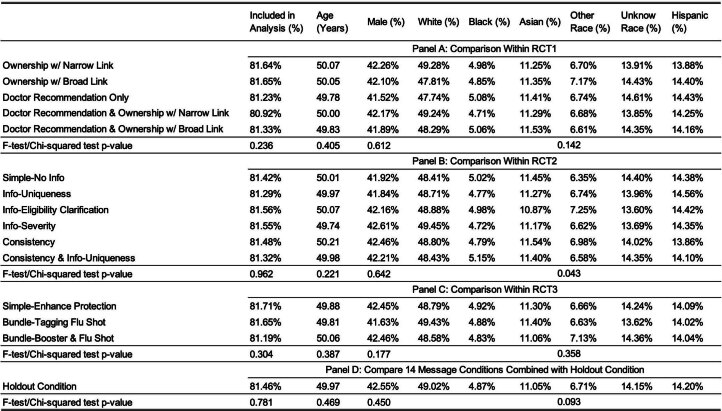
Randomization Check of the RCTs

This table compares participant demographics across message conditions within each RCT (Panels A-C) as well as between the message conditions combined and the aggregate holdout condition (Panel D). The variables reported here include whether a participant that was initially enrolled in the RCTs met the eligibility criteria to be included in our final analysis sample; participant age; whether a participant was a man (versus a woman, other or unknown); and whether a participant’s race/ethnicity on their medical record was White, Black, Asian, other races, unknown, or Hispanic/Latino. For age, gender, and race/ethnicity variables, this table only reports information about patients included in the analysis sample. For whether patients were retained in the analysis, patient age, and whether patients were male, we predicted each variable using OLS regressions as a function of indicators for the relevant conditions under comparison in each panel, with robust standard errors to correct for heterogeneity. A two-sided *F*-test was conducted for each regression to compare the overall significance of differences across relevant conditions for a given variable. To summarize across all categories of race/ethnicity, we ran a chi-squared test to assess whether there was any association between randomly assigned conditions in each panel and race/ethnicity.

## Data Availability

The data analysed in this article about RCTs were provided by UCLA Health and contain protected health information. To protect participant privacy, we cannot publicly post individual-level data. Upon request to the corresponding authors, and approval by the UCLA Health Data Oversight committee, qualified researchers can obtain access to the deidentified data about these trials. A formal contract will be signed and an independent data protection agency should oversee the sharing process to ensure the safety of the data. Data about all our surveys are available at ref. ^50^.

## Code availability

The code to replicate the analyses and figures in Main and Supplementary Information is available at ref. ^50^.
