# Supplementary material for: Field testing the transferability of behavioural science knowledge on promoting vaccinations
Source: Nat Hum Behav. 2024 Mar 14;8(5):878–90. doi: 10.1038/s41562-023-01813-4 (PMC11132983; doi:10.1038/s41562-023-01813-4)
Supplement: Supplementary file 1 — Supplementary Tables 1–10, Methods and Notes. [file 41562_2023_1813_MOESM1_ESM.pdf]

nature human behaviour

Article https://doi.org/10.1038/s41562-023-01813-4

Field testing the transferability of behavioural science knowledge on promoting vaccinations

In the format provided by the authors and unedited

Supplementary Information

Supplementary Table 1. Megastudy Approach to Comparing Booster Uptake Between Conditions Across Three RCTs

	Booster Uptake			
	(1)	(2)	(3)	(4)
Ownership w/ Narrow Link	0.019<br>(<0.001)<br>[0.014,0.025]			
Ownership w/ Broad Link	0.013<br>(<0.001)<br>[0.007,0.018]			
Doctor Recommendation Only	0.013<br>(<0.001)<br>[0.008,0.019]			
Doctor Rec & Ownership w/ Narrow Link	0.019<br>(<0.001)<br>[0.013,0.025]			
Doctor Rec & Ownership w/ Broad Link	0.015<br>(<0.001)<br>[0.009,0.021]			
Simple-No Info	0.009<br>(0.002)<br>[0.003,0.014]			
Info-Uniqueness	0.015<br>(<0.001)<br>[0.009,0.021]			
Info-Eligibility Clarification	0.011<br>(<0.001)<br>[0.006,0.017]			
Info-Severity	0.009<br>(0.002)<br>[0.003,0.014]			
Consistency	0.008<br>(0.008)<br>[0.002,0.013]			
Consistency & Info-Uniqueness	0.010<br>(0.001)<br>[0.004,0.015]			
Simple-Enhance Protection	0.007<br>(0.008)<br>[0.002,0.013]			
Bundle-Tagging Flu Shot	0.007<br>(0.010)<br>[0.002,0.013]			
Bundle-Booster & Flu Shot	0.003<br>(0.325)<br>[-0.003,0.008]			
Text Reminder		0.011<br>(<0.001)<br>[0.008,0.014]		
Containing Ownership			0.008<br>(<0.001)<br>[0.005,0.011]	
Containing Ownership w/o Doctor Rec				0.008<br>(0.002)<br>[0.003,0.013]
Control Variables	Yes	Yes	Yes	Yes
N	314,824	314,824	240,775	74,235
R^2	0.018	0.018	0.018	0.018
Degrees of Freedom	314,769	314,782	240,733	74,193
Conditions Included	All conditions	All conditions	All message conditions with link	Ownership w/ Narrow Link<br>Ownership w/ Broad Link<br>Simple-No Info<br>Simple-Enhance Protection
Reference Group	Holdout Condition	Holdout Condition	Messages w/o ownership framing	Simple-No Info<br>Simple-Enhance Protection
Mean in the Reference Group	12.39%	12.39%	13.27%	13.20%

Legend: The table reports ordinary least squares (OLS) regressions predicting whether patients received the bivalent booster within four weeks of their assigned message date. The key predictors in Column 1 are binary indicators of the 14 message conditions. In Column 2, *Text Reminder* is coded as 1 when participants received any text message and 0 otherwise. In Column 3, *Containing Ownership* is coded as 1 when participants received any message with the ownership language and 0 when participants received other messages that contained links without the ownership language. In Column 4, *Containing Ownership w/o Doctor Rec* is coded as 1 when participants received the *Ownership w/ Narrow Link* or the *Ownership w/ Broad Link* message and 0 when participants received the *Simple-No Info* or the *Simple-Enhance Protection* message. Control variables include preregistered covariates of gender (male, female, with people whose gender was "other" or unknown to us as the reference group), age, race/ethnicity (Hispanic, White non-Hispanic, Black non-Hispanic, Asian non-Hispanic, other/mixed, with people whose race was unknown to us and whose ethnicity was not Hispanic as the reference group), as well as indicators for the 33 time slots (i.e., 3 times across 11 days) that patients were randomly assigned to. We report point estimates, exact p-values based on two-sided tests in parentheses, and 95% confidence intervals in brackets.

See https://osf.io/qhw95 for adjusted p-values in Column 1 after correcting for 14 comparisons of individual messages with the holdout.

Supplementary Table 2. Regression-estimated Differences in Booster Uptake Between Message Conditions Within the First RCT

	Booster Uptake	
	(1)	(2)
Ownership Language w/ Link	0.003	
	(0.371)	
	[-0.003,0.009]	
Doctor Recommendation &	0.004	
Ownership Language w/ Link	(0.233)	
	[-0.002,0.010]	
Narrow Link Only		0.005
		(0.043)
		[0.000,0.010]
Control Variables	Yes	Yes
N	92,507	74,033
R^2	0.018	0.018
Degrees of Freedom	92,464	73,991
	Doctor Recommendation & Ownership Language w/ Link vs. Ownership Language w/ Link: B = 0.001, p = 0.715, 95% CI = [-0.004, 0.006]	
Conditions Included	All message conditions in RCT1	Four message conditions with link in RCT1
Reference Group	Doctor Recommendation Only	Ownership w/ Broad Link
		Doctor Rec & Ownership w/ Broad Link
Mean in the Reference Group	13.66%	13.77%

Legend: The table reports ordinary least squares (OLS) regressions predicting whether patients received the bivalent booster within four weeks of their assigned message date. *Ownership Language with Link* is coded as 1 when participants received the *Ownership w/ Narrow Link* or *Ownership w/ Broad Link* message and 0 otherwise. *Doctor Recommendation & Ownership Language with Link* is coded as 1 when participants received the *Doctor Recommendation & Ownership w/ Narrow Link* or the *Doctor Recommendation & Ownership w/ Broad Link* message and 0 otherwise. *Narrow Link Only* is coded as 1 when participants received the *Ownership w/ Narrow Link* or *Doctor Recommendation & Ownership w/ Narrow Link* message and 0 otherwise. Control variables include preregistered covariates of gender (male, female, with people whose gender was "other" or unknown to us as the reference group), age, race/ethnicity (Hispanic, White non-Hispanic, Black non-Hispanic, Asian non-Hispanic, other/mixed, with people whose race was unknown to us and whose ethnicity was not Hispanic as the reference group), as well as indicators for the 33 time slots (i.e., 3 times across 11 days) that patients were randomly assigned to. We report point estimates, exact p-values based on two-sided tests in parentheses, and 95% confidence intervals in brackets.

Supplementary Table 3. Regression-estimated Differences in Perceived Persuasiveness and Booster Intentions Between RCT2 Messages in an Online Experiment

	Perceived Persuasiveness			Booster Intentions		
	(1)	(2)	(3)	(4)	(5)	(6)
Info-Uniqueness	0.414		0.801	0.321		0.560
	(0.002)		(<0.001)	(0.052)		(0.001)
	[0.149,0.679]		[0.539,1.063]	[-0.003,0.645]		[0.242,0.877]
Info-Severity	0.383		0.775	0.158		0.404
	(0.004)		(<0.001)	(0.342)		(0.013)
	[0.122,0.645]		[0.516,1.033]	[-0.167,0.483]		[0.085,0.723]
Information Provision		0.661			0.409	
		(<0.001)			(0.002)	
		[0.445,0.877]			[0.147,0.671]	
Info-Eligibility Clarification			0.392			0.251
			(0.004)			(0.131)
			[0.124,0.660]			[-0.075,0.578]
Consistency		0.326	0.325		0.385	0.385
		(0.014)	(0.014)		(0.019)	(0.019)
		[0.066,0.586]	[0.065,0.586]		[0.064,0.706]	[0.064,0.705]
Consistency & Info-Uniqueness		0.913	0.912		0.417	0.416
		(<0.001)	(<0.001)		(0.013)	(0.013)
		[0.648,1.178]	[0.647,1.178]		[0.088,0.746]	[0.088,0.745]
Control Variables	Yes	Yes	Yes	Yes	Yes	Yes
N	895	1,774	1,774	895	1,774	1,774
R^2	0.026	0.047	0.053	0.007	0.009	0.011
Degrees of Freedom	884	1,762	1,760	884	1,762	1,760
F-test of Joint Significance	F(2, 884) = 5.78, p = 0.003			F(2, 884) = 1.89, p = 0.152		
Conditions Included	Info-Uniqueness Info-Eligibility Info-Severity	All conditions	All conditions	Info-Uniqueness Info-Eligibility Info-Severity	All conditions	All conditions
Reference Group	Info-Eligibility	Simple-No Info	Simple-No Info	Info-Eligibility	Simple-No Info	Simple-No Info
Mean in the Reference Group	4.03	3.64	3.64	4.23	3.98	3.98

Legend: The table reports ordinary least squares (OLS) regressions predicting the perceived persuasiveness of a message (Columns 1-3) and participants' intentions to get the bivalent booster after reading the message (Columns 4-6). Both outcome measures were assessed on a 7-point Likert scale. *Info-Uniqueness*, *Info-Severity*, *Info-Eligibility Clarification*, *Consistency*, and *Consistency & Info-Uniqueness* are binary indicators that equal 1 for participants who received the corresponding message and 0 otherwise. *Information Provision* is coded as 1 when participants received one of the three information-provision messages (*Info-Uniqueness*, *Info-Severity*, *Info-Eligibility Clarification*) and 0 otherwise. Control variables include preregistered covariates of gender (male, female, with people whose gender was "other" or unknown to us as the reference group), age, race/ethnicity (Hispanic, White non-Hispanic, Black non-Hispanic, Asian non-Hispanic, with people whose race was other, mixed, or unknown to us and whose ethnicity was not Hispanic as the reference group), and an indicator for missing demographics. We report point estimates, exact p-values based on two-sided tests in parentheses, and 95% confidence intervals in brackets.

Findings from Columns 2 and 5 are reported in the paper. See https://osf.io/qhw95 for adjusted p-values after correcting for three comparisons with the Simple-No Reminder message.

Supplementary Table 4. Regression-estimated Differences in Booster Uptake Between Message Conditions within the Second RCT

	Booster Uptake	
	(1)	(2)
Info-Uniqueness	0.003	
	(0.345)	
	[-0.004,0.010]	
Info-Severity	-0.003	
	(0.439)	
	[-0.010,0.004]	
Information Provision		0.003
		(0.297)
		[-0.003,0.009]
Consistency		-0.001
		(0.732)
		[-0.008,0.006]
Consistency & Info-Uniqueness		0.001
		(0.781)
		[-0.006,0.008]
Control Variables	Yes	Yes
N	55,584	111,126
R^2	0.017	0.018
Degrees of Freedom	55,541	111,082
F-test of Joint Significance	$F(2, 55{,}541) = 1.48$, $p = 0.228$	
Conditions Included	Info-Uniqueness<br>Info-Eligibility<br>Info-Severity	All message conditions in RCT2
Reference Group	Info-Eligibility	Simple-No Info
Mean in the Reference Group	13.53%	13.27%

Legend: The table reports ordinary least squares (OLS) regressions predicting whether patients received the bivalent booster within four weeks of their assigned message date. *Info-Uniqueness*, *Info-Severity*, *Consistency*, and *Consistency & Info-Uniqueness* are binary indicators that equal 1 for participants who received the corresponding message and 0 otherwise. *Information Provision* is coded as 1 when participants received one of the three information-provision messages (*Info-Uniqueness*, *Info-Severity*, *Info-Eligibility Clarification*) and 0 otherwise. Control variables include preregistered covariates of gender (male, female, with people whose gender was "other" or unknown to us as the reference group), age, race/ethnicity (Hispanic, White non-Hispanic, Black non-Hispanic, Asian non-Hispanic, other/mixed, with people whose race was unknown to us and whose ethnicity was not Hispanic as the reference group), as well as indicators for the 33 time slots (i.e., 3 times across 11 days) that patients were randomly assigned to. We report point estimates, exact p-values based on two-sided tests in parentheses, and 95% confidence intervals in brackets.

Supplementary Table 5. Regression-estimated Differences in Perceived Persuasiveness, Booster Intentions, and Perceived Convenience Between RCT3 Messages in Online Experiments

	(1)	(2)	(3)	(4)	(5)	(6)	(7)	(8)	(9)
	Perceived Persuasiveness			Perceived Convenience			Booster Intentions		
Bundle-Tagging Flu Shot	-0.353		0.260	0.102		1.805	0.136		0.171
	(0.004)		(0.031)	(0.437)		(0.000)	(0.380)		(0.241)
	[-0.592,-0.115]		[0.023,0.497]	[-0.156,0.360]		[1.511,2.098]	[-0.168,0.439]		[-0.115,0.458]
Bundle-Booster & Flu Shot			0.597			1.692			0.020
			(0.000)			(0.000)			(0.897)
			[0.360,0.834]			[1.399,1.986]			[-0.278,0.317]
Bundle		0.427			1.749			0.096	
		(0.000)			(0.000)			(0.451)	
		[0.222,0.633]			[1.485,2.012]			[-0.154,0.346]	
Control Variables	Yes	Yes	Yes	Yes	Yes	Yes	Yes	Yes	Yes
N	649	989	989	565	860	860	649	989	989
R^2	0.036	0.039	0.046	0.038	0.200	0.201	0.020	0.014	0.015
Degrees of freedom	639	979	978	555	850	849	639	979	978
Conditions Included	Two bundling conditions	All conditions	All conditions	Two bundling conditions	All conditions	All conditions	Two bundling conditions	All conditions	All conditions
Reference Group	Bundle-Booster & Flu Shot	Simple-Enhance Protection	Simple-Enhance Protection	Bundle-Booster & Flu Shot	Simple-Enhance Protection	Simple-Enhance Protection	Bundle-Booster & Flu Shot	Simple-Enhance Protection	Simple-Enhance Protection
Mean in the Reference Group	4.523	3.935	3.935	5.449	3.749	3.749	4.214	4.185	4.185

Legend: The table reports ordinary least squares (OLS) regressions predicting the perceived persuasiveness of a message (Columns 1-3), perceived convenience of getting the flu shot at the same time as the bivalent booster (Columns 4-6), and participants' intentions to get the bivalent booster (Columns 7-9). These outcome measures were assessed on a 7-point Likert scale. *Bundle-Tagging Flu Shot* is coded as 1 when participants received the *Bundle-Tagging Flu Shot* message and 0 otherwise. *Bundle-Booster & Flu Shot* is coded as 1 when participants received the *Bundle-Booster & Flu Shot* message and 0 otherwise. *Bundle* is coded as 1 when participants received one of the two vaccine bundling messages (*Bundle-Tagging Flu Shot*, *Bundle-Booster & Flu Shot*) and 0 otherwise. Control variables include preregistered covariates of gender (male, female, with people whose gender was "other" or unknown to us as the reference group), age, race/ethnicity (Hispanic, White non-Hispanic, Black non-Hispanic, Asian non-Hispanic, with people whose race was other, mixed, or unknown to us and whose ethnicity was not Hispanic as the reference group), and an indicator for missing demographics. We report point estimates, exact p-values based on two-sided tests in parentheses, and 95% confidence intervals in brackets.

Supplementary Table 6. Regression-estimated Differences in Booster Uptake Between Message Conditions Within the Third RCT

	Booster Uptake	
	(1)	(2)
Bundle-Tagging Flu Shot	0.005	
	(0.186)	
	[-0.002,0.011]	
Bundle		-0.002
		(0.411)
		[-0.008,0.003]
Control Variables	Yes	Yes
N	37,034	55,616
R^2	0.019	0.019
Degrees of Freedom	36,992	55,574
Conditions Included	Bundle-Tagging Flu Shot<br>Bundle-Booster & Flu Shot	All message conditions in RCT3
Reference Group	Bundle-Booster & Flu Shot	Simple-Enhance Protection
Mean in the Reference Group	12.66%	13.13%

Legend: The table reports ordinary least squares (OLS) regressions predicting whether patients received the bivalent booster within four weeks of their assigned message date. *Bundle-Tagging Flu Shot* is coded as 1 when participants received the *Bundle-Tagging Flu Shot* message and 0 otherwise. *Bundle* is coded as 1 when participants received one of the two vaccine bundling messages (*Bundle-Tagging Flu Shot*, *Bundle-Booster & Flu Shot*) and 0 otherwise. Control variables include preregistered covariates of gender (male, female, with people whose gender was "other" or unknown to us as the reference group), age, race/ethnicity (Hispanic, White non-Hispanic, Black non-Hispanic, Asian non-Hispanic, other/mixed, with people whose race was unknown to us and whose ethnicity was not Hispanic as the reference group), as well as indicators for the 33 time slots (i.e., 3 times across 11 days) that patients were randomly assigned to. We report point estimates, exact p-values based on two-sided tests in parentheses, and 95% confidence intervals in brackets.

Supplementary Table 7. Exploratory Analyses of Heterogeneous Treatment Effect of Receiving a Text Reminder by Proxies of Socioeconomic Status

	Booster Uptake		
	(1)	(2)	(3)
Text Reminder	0.011<br>(<0.001)<br>[0.007,0.014]	0.011<br>(<0.001)<br>[0.007,0.014]	0.011<br>(<0.001)<br>[0.007,0.014]
SVI Above 50	-0.016<br>(<0.001)<br>[-0.022,-0.010]		
Text Reminder X SVI Above 50	0.002<br>(0.552)<br>[-0.005,0.009]		
Neighborhood Median Household Income Below 25th Percentile		-0.013<br>(<0.001)<br>[-0.019,-0.007]	
Text Reminder X Neighborhood Median Household Income Below 25th Percentile		0.002<br>(0.510)<br>[-0.005,0.009]	
Neighborhood Bachelor Rate Below 25th Percentile			-0.030<br>(<0.001)<br>[-0.036,-0.024]
Text Reminder X Neighborhood Bachelor Rate Below 25th Percentile			0.003<br>(0.442)<br>[-0.004,0.009]
Control Variables	Yes	Yes	Yes
N	310,979	305,365	305,451
R^2	0.018	0.019	0.020
Degrees of Freedom	310,935	305,321	305,407
Conditions Included	All conditions across three RCTs	All conditions across three RCTs	All conditions across three RCTs
Reference Group	Holdout Condition	Holdout Condition	Holdout Condition

Legend: The table reports ordinary least squares (OLS) regressions predicting whether patients received the bivalent booster within four weeks of their assigned message date. *Text Reminder* is coded as 1 when participants received any text message and 0 otherwise. *SVI Above 50* equals 1 for patients whose social vulnerability index (SVI) based on UCLA Health's records was above 50 and 0 otherwise. *Neighborhood Median Household Income Below* 25th *Percentile* equals 1 for patients whose neighborhood median household income based on 2015-2019 estimates was below $71,400 and 0 otherwise. *Neighborhood Bachelor Rate Below* 25th *Percentile* equals 1 for patients whose neighborhood bachelor rate within residents aged 25 and older was below 35% and 0 otherwise. Columns 1, 2, and 3 only include patients for whom we have information about their SVI, the median household income in their neighborhood (based on five-digit zip codes), and the percentage of residents aged 25 and older with a bachelor's degree in their neighborhood (based on five-digit zip codes), respectively. Control variables include gender (male, female, with people whose gender was "other" or unknown to us as the reference group), age, race/ethnicity (Hispanic, White non-Hispanic, Black non-Hispanic, Asian non-Hispanic, other/mixed, with people whose race was unknown to us and whose ethnicity was not Hispanic as the reference group), as well as indicators for the 33 time slots (i.e., 3 times across 11 days) that patients were randomly assigned to. We report point estimates, exact p-values based on two-sided tests in parentheses, and 95% confidence intervals in brackets.

Supplementary Table 8. Randomization of Patients into Dates and Times Across Three RCTs

	Time			
**Date**	9:00 A.M.	12:00 P.M.	4:00 P.M.	Total
10/18/2023	11,560 (9,757)	11,560 (9,822)	11,560 (9,857)	34,680 (29,436)
10/19/2023	11,560 (9,725)	11,560 (9,683)	11,560 (9,765)	34,680 (29,173)
10/20/2023	11,560 (9,550)	11,560 (9,628)	11,560 (9,627)	34,680 (28,805)
10/21/2023	11,560 (9,550)	11,560 (9,598)	11,560 (9,564)	34,680 (28,712)
10/24/2023	12,134 (9,954)	12,133 (9,932)	12,129 (9,968)	36,396 (29,854)
10/25/2023	12,130 (9,819)	12,127 (9,911)	12,133 (9,885)	36,390 (29,615)
10/26/2023	12,131 (9,841)	12,129 (9,788)	12,129 (9,849)	36,389 (29,478)
10/27/2023	11,560 (9,254)	11,560 (9,334)	11,560 (9,259)	34,680 (27,847)
10/28/2023	11,560 (9,259)	11,560 (9,154)	11,560 (9,183)	34,680 (27,596)
10/31/2023	11,560 (9,048)	11,560 (9,051)	11,560 (9,077)	34,680 (27,176)
11/1/2023	11,560 (9,024)	11,560 (9,079)	11,560 (9,029)	34,680 (27,132)
Total	128,875 (104,781)	128,869 (104,980)	128,871 (105,063)	386,615 (314,824)

Note. In each cell, the first number refer to the number of patients who were assigned to each date and time, and the number in parentheses refers to the number of patients who satisfied the preregistered inclusion/exclusion criteria for data analysis (as described in Methods) and thus were used in data analysis.

Supplementary Table 9. Compare Booster-Related Beliefs and Perceptions by Booster Uptake Intentions

Booster Intentions	No		Uncertain		Yes		Compare "Yes" vs. "Uncertain"/"No"
	Mean	SD	Mean	SD	Mean	SD	p-value of two-sided t-test, 95% CI of the difference, Cohen's d
Perceived Eligibility	2.74	0.45	2.50	0.50	2.80	0.43	$p < 0.001$, 95% CI = [0.17, 0.33], d = 0.55
Infection Likelihood without Booster	30.62	19.53	34.64	19.43	45.92	23.62	$p < 0.001$, 95% CI = [8.23, 16.11], d = 0.55
Booster Effectiveness for Infections	1.14	15.03	9.30	15.76	20.60	20.27	$p < 0.001$, 95% CI = [9.76, 16.44], d = 0.70
Long COVID Likelihood without Booster	22.14	20.69	33.82	22.61	42.47	25.06	$p < 0.001$, 95% CI = [6.92, 15.52], d = 0.46
Booster Effectiveness for Long COVID	3.33	11.73	11.83	17.18	24.28	22.47	$p < 0.001$, 95% CI = [10.68, 17.97], d = 0.70
Infection Severity without Booster	3.67	2.20	4.27	1.68	5.34	1.82	$p < 0.001$, 95% CI = [0.88, 1.52], d = 0.66
Booster Effectiveness for Infection Severity	0.64	1.32	1.48	1.41	2.80	1.82	$p < 0.001$, 95% CI = [1.20, 1.80], d = 0.88
Comparative Efficacy over Original Booster	4.83	2.50	5.58	1.71	6.11	2.35	$p < 0.001$, 95% CI = [0.30, 1.08], d = 0.31
Guideline Confusion	5.38	2.55	5.22	2.21	3.72	2.79	$p < 0.001$, 95% CI = [1.08, 2.01], d = 0.59
Perceived Doctor Recommendation	68.60	21.32	70.14	20.80	82.58	16.58	$p < 0.001$, 95% CI = [9.55, 16.02], d = 0.70
Number of participants	42		149		342		

Legend: The table reports the mean value and standard deviation for a series of measures of perceptions and beliefs about COVID-19 and the bivalent booster, for participants who chose either "I have already gotten the COVID-19 bivalent booster" or "I plan to get the COVID-19 bivalent booster but I have not done it yet" (*Yes*), participants who chose "I am not sure if I will get the COVID-19 bivalent booster" (*Uncertain*), and participants who chose "I do not plan to get the COVID-19 bivalent booster" (*No*). The original scale of *Infection Likelihood without Booster*, *Long COVID Likelihood without Booster*, and *Perceived Doctor Recommendation* was 0% to 100%, and we multiply the raw values by 100 for ease of presentation. The original scale of *Booster Effectiveness for Infections* and *Booster Effectiveness for Long COVID* was -100% to 100%, and we multiply the raw values by 100 for ease of presentation. *Perceived Eligibility* is coded such that 3 equals "Yes, I believe I am eligible", 2 equals "I am not sure", and 1 equals "No, I don't believe I am eligible." The last column reports the p-value of two-sided two-sample t-test that compares each variable between "Yes" participants and "Uncertain" or "No" participants, along with the 95% CI of the difference and the corresponding Cohen's d. The degrees of freedom is 531 for the two-sided two-sample t-test for all variables.

Supplementary Table 10. Match Condition Names in the Main Text with Condition Names in the Preregistrations

Panel A: Three RCTs

**RCT # and Clinicaltrials #**	**Condition Name in the Main Text**	**Condition Name in the Corresponding Preregistration**
RCT1 https://clinicaltrials.gov/ct2/show/NCT05586204	Ownership w/ Narrow Link	Facilitate Action Narrowly
	Ownership w/ Broad Link	Facilitate Action Broadly
	Doctor Recommendation Only	Boost Intentions Only
	Doctor Recommendation & Ownership w/ Narrow Link	Boost Intentions and Facilitate Action Narrowly
	Doctor Recommendation & Ownership w/ Broad Link	Boost Intentions and Facilitate Action Broadly
RCT2 https://clinicaltrials.gov/ct2/show/NCT05586178	Simple-No Info	Simple Reminder
	Info-Uniqueness	Uniqueness Information Reminder
	Info-Eligibility Clarification	Eligibility Information Reminder
	Info-Severity	Severity Information Reminder
	Consistency	Consistency Reminder
	Consistency & Info-Uniqueness	Consistency and Uniqueness Information Reminder
RCT3 https://clinicaltrials.gov/ct2/show/NCT05586165	Simple-Enhance Protection	Simple Reminder
	Bundle-Tagging Flu Shot	Flu Tag Along
	Bundle-Booster & Flu Shot	Covid-19 Booster + Flu Bundle

Panel B: Online Experiments

**Experiment & Preregistration URL**	**Condition Name in the Main Text**	**Condition Name in the Corresponding Preregistration**
Online Experiment accompanying RCT2 https://aspredicted.org/blind.php?x=MYX_HZH	Simple-No Info	Simple
	Info-Uniqueness	Uniqueness Information
	Info-Eligibility Clarification	Eligibility Information
	Info-Severity	Severity Information
	Consistency	Consistency
	Consistency & Info-Uniqueness	Consistency and Uniqueness Information
Online Experiments accompanying RCT3 https://aspredicted.org/blind.php?x=DP1_DDN https://aspredicted.org/L17_JW2	Simple-Enhance Protection	Simple Reminder
	Bundle-Tagging Flu Shot	Flu Tag Along
	Bundle-Booster & Flu Shot	Flu and COVID-19 Bundle

Supplementary Methods

Randomized Controlled Trials

Information about Links in the Text Messages

For CVS Pharmacy, the link was https://www.cvs.com/vaccine/intake/store/schedule-options. For the general website, we used a link that preselected Moderna and Pfizer-BioNTech bivalent boosters as vaccine options, so as to further reduce the steps patients had to take to schedule the appointment (https://www.vaccines.gov/search/?medicationGuids=018b22d2-054b-4d42-8279-d4efb511aec6%2C25e72c3c-8f6c-4738-9638-08911a400e70&appointments=true&medicationKeys=pfizer_covid_19_vaccine_bivalent_booster%2Cmoderna_covid_19_vaccine_bivalent_booster). Text messages sent out on the first three days of the RCTs also included a link to UCLA Health: https://my.uclahealth.org/MyChart/Scheduling. When sending out text messages, the vendor replaced the exact URLs with shorter personalized links in order to reduce the length of the text messages and track whether each patient clicked a given link. The personalized links had the same format, starting from https://well.app.

Megastudy Analysis & Analysis of RCT1

In our preregistration for RCT1 (https://clinicaltrials.gov/ct2/show/NCT05586204), we indicated that in addition to examining the research questions that individual RCTs were designed to test, we would also report results across the three RCTs in a megastudy fashion following Milkman et al. (2021).

To estimate the effect of receiving each of the reminders (relative to the holdout condition), we ran an OLS regression with heteroskedasticity-robust standard errors to predict Booster Uptake as a function of the 14 indicators for random assignment to each of the 14 message arms (with the holdout condition as the reference group). The regression coefficients are reported in Column 1 of Supplementary Table 1. To estimate the average effect of receiving a text reminder across three RCTs, we ran another OLS regression to predict Booster Uptake as a function of the Text Reminder indicator, which equals one for any of the message arms and zero for the holdout condition. See Column 2 of Supplementary Table 1 for the regression results.

We conducted two exploratory analyses to estimate the effect of adding the ownership language to reminders with links. First, we compared the four text messages containing the "claim your dose" language and links with all the other messages that contained links without the ownership language. We ran an OLS regression to predict Booster Uptake as a function of a binary indicator, Containing Ownership, which equals one for the four conditions that included the "claim your dose" language and zero for other message conditions that contained links. Second, in an OLS regression, we compared the two text messages that only contained the "claim your dose" language without highlighting doctor recommendations (i.e., Ownership w/ Narrow Link and Ownership w/ Broad Link) with the simple reminders in the second and third RCTs (i.e., Simple-No Info and Simple-Enhance Protection). The key predictor in this regression is a binary indicator, Containing Ownership w/o Doctor Recommendation, which equals one for the former two conditions and zero for the latter two conditions. Note that Ownership w/ Narrow Link and Simple-Enhance Protection messages had the same (narrow) link, and Ownership w/ Broad Link and Simple-No Info had the same (broad) link. Results of these analyses are shown in Columns 3 and 4 of Supplementary Table 1.

Leveraging data from RCT1 alone, we examine the value of simultaneously a) highlighting doctor recommendations and b) providing an appointment scheduling link and

prompting people to claim their dose right away, relative to independently administering these strategies. Following the preregistration, we constructed two binary indicators: Ownership Language w/ Link (which equals one for Ownership w/ Narrow Link and Ownership w/ Broad Link messages and zero otherwise) and Doctor Recommendation & Ownership Language w/ Link (which equals one for Doctor Recommendation & Ownership w/ Narrow Link and Doctor Recommendation & Ownership w/ Broad Link messages and zero otherwise). In an OLS regression that involved all five message conditions in the first RCT, we predicted Booster Uptake with the Ownership Language w/ Link indicator and the Doctor Recommendation & Ownership Language w/ Link indicator. In this regression, the Doctor Recommendation Only message was the reference group. The results of this regression are shown in Column 1 of Supplementary Table 2. The coefficient on Doctor Recommendation & Ownership Language w/ Link captures the regression-estimated effect of adding the ownership language and appointment scheduling link to a message that only highlighted doctor recommendation. Comparing the coefficient on Ownership Language w/ Link with the coefficient on Doctor Recommendation & Ownership Language w/ Link allows us to estimate the effect of adding doctor recommendation to a message that only contained the ownership language and appointment scheduling link.

To examine whether it is better to provide people with a link to one vaccination venue or a link to a variety of vaccination venues, we compared the two conditions that contained only a "narrow" link to CVS Pharmacy (Ownership w/ Narrow Link and Doctor Recommendation & Ownership w/ Narrow Link) with the two conditions that replaced the CVS Pharmacy link with a "broad" link to www.vaccines.org (Ownership w/ Broad Link and Doctor Recommendation & Ownership w/ Broad Link). In an OLS regression involving only these four message conditions in the first RCT, we predicted Booster Uptake with the binary indicator of Narrow Link Only (which equals one for the Ownership w/ Narrow Link and Doctor Recommendation & Ownership w/ Narrow Link conditions and zero for the Ownership w/ Broad Link and Doctor Recommendation & Ownership w/ Broad Link conditions). The regression results are reported in Column 2 of Supplementary Table 2.

Analysis of RCT2

Following the preregistration, we began with a comparison of three information-provision messages to determine whether we should collapse them into one condition. We predicted Booster Uptake as a function of an indicator of the Info-Uniqueness Message and an indicator for the Info-Severity message (with the Info-Eligibility Clarification message as the reference group). This regression only included observations from these three information-provision conditions. As shown in Supplementary Table 4 Column 1, a joint significance F-test fails to reject the null hypothesis that the three information-provision message conditions had the same true value of booster uptake rates ($F(2, 55{,}541) = 1.48$, $p = 0.23$). As a result, we collapsed the three information-provision message conditions when we estimated the effect of adding these information-provision strategies to a basic reminder. In an OLS regression that included all five message conditions in RCT2, we predicted Booster Uptake with (1) a binary indicator, Information Provision, which equals one if a patient received one of the three information-provision messages and zero if the patient received the Simple-No Info message; (2) a binary indicator for getting the Consistency message; and (3) a binary indicator for getting the Consistency & Info-Uniqueness message. The Simple-No Info message was the reference group in this regression. The results are reported in Supplementary Table 4 Column 2.

Analysis of RCT3

Following the preregistration, we first compared the two vaccine bundling messages that referenced the flu shot to determine whether we should collapse them into one condition. We predicted Booster Uptake with an indicator of whether patients received the Bundle-Tagging Flu Shot message (or the Bundle-Booster & Flu Shot message, which was the reference group in this regression). As shown in Column 1 in Supplementary Table 6, these two messages did not significantly differ from each other (B = 0.0046, p = 0.19, 95% CI = [-0.0022, 0.0113]). Thus, we followed the preregistration to collapse these two conditions and compare them together with the Simple-Enhance Protection condition to examine whether reminding patients about the convenience of getting the flu shot at the same time as the booster could increase booster uptake. In an OLS regression including all three message conditions in RCT3, we predicted Booster Uptake with a binary indicator, Bundling, which equals one if a patient received one of the bundling messages and zero if the patient received the Simple-Enhance Protection message. The results are reported in Supplementary Table 6 Column 2.

Exploratory Analyses: Heterogenous Treatment Effect of Receiving a Reminder

Following the recommendation of one reviewer, we conducted three sets of exploratory analyses to see whether the average effect of reminders differed between patients with relatively low socioeconomic status and those with relatively high socioeconomic status.

First, for patients in our sample, UCLA Health categorized them into two buckets based on whether their social vulnerability index (SVI) was below 50 or above 50, with the latter representing on average greater social vulnerability than the former. Among patients with a known SVI in our analysis sample (n = 310,979), about 75% of them had an SVI below 50, and 25% had an SVI above 50. In an OLS regression involving patients with a known SVI, we predicted Booster Uptake as a function of the Text Reminder indicator (which equals one for any of the message arms and zero for the holdout condition), the SVI Above 50 indicator (which equals one for patients whose SVI was above 50 and zero otherwise), and their interaction, along with other covariates mentioned in the Methods section.

Second, we matched patients' five-digit zip code with the 2019 American Community Survey 5-Year Estimates (available at https://data.census.gov/) to obtain the median household income in their neighborhood based on 2015-2019 estimates. Among patients in our analysis sample with a valid zip code in California that could be matched with income data (n = 305,365), the 25th percentile of the distribution of neighborhood median household income was $71,400. In an OLS regression involving patients with a value of neighborhood median household income, we predicted Booster Uptake as a function of the Text Reminder indicator, the Neighborhood Median Household Income Below 25th Percentile indicator (which equals one for patients whose neighborhood median household income was below $71,400 and zero otherwise), and their interaction, along with other covariates mentioned in the Methods section.

Third, we matched patients' five-digit zip code with the 2019 American Community Survey 5-Year Estimates (available at https://data.census.gov/) to obtain the population aged 25 and older and the population in this age group with a bachelor's degree or higher in their neighborhood based on 2015-2019 estimates. Using these data points, we calculated the percentage of residents aged 25 and above with at least a bachelor's degree in each patient's neighborhood. Among patients with a value for this variable (n = 305,451), the 25th percentile of the distribution of neighborhood bachelor rate within residents aged 25 and older was 35%. For reference, the national percentage of people aged 25 and older who had completed a bachelor's degree or higher was 37.9% in 2021 in the United States (https://www.pewresearch.org/short-reads/2022/04/12/10-facts-about-todays-college-graduates). In an OLS regression involving

patients with a value of neighborhood bachelor rate, we predicted Booster Uptake as a function of the Text Reminder indicator, the Neighborhood Bachelor Rate Below 25th Percentile indicator (which equals one for patients whose neighborhood bachelor rate within residents aged 25 and older was below 35% and zero otherwise), and their interaction, along with other covariates mentioned in the Methods section.

As shown in Supplementary Table 7, the average effect of receiving a reminder in our RCTs did not statistically significantly differ between patients with relatively low socioeconomic status (i.e., patients whose social vulnerability index was above 50 and among the top 25% in our sample, patients living in neighborhood with median household income among the bottom 25% of the sample, or patients for whom the percentage of neighbors aged 25 and older who had at least a bachelor's degree was among the bottom 25% of the sample) and patients with relatively high socioeconomic status.

Online Experiment Examining Messages Tested in RCT2

Our data collection spanned four consecutive days. In the first three days of data collection, per our preregistration, participants were asked at the start of the survey (1) whether they had completed the primary COVID-19 vaccine series and (2) whether they had gotten a COVID-19 vaccine in the past 2 months. We aimed to exclude people who had not completed the primary series (because they were not eligible for the bivalent booster) as well as those that obtained a dose within two months (because they either were not eligible for the bivalent booster at the time of the study or they had already received it). Participants were unaware of these selection criteria. The data collection was getting slow during the third day, and the rate at which people reported getting a COVID-19 vaccine dose within the past two months was higher than our expectations. Since we thought participants may have failed to notice we were asking them about COVID-19 vaccines obtained in a specific time window (i.e., in the past two months), we changed the screening question during the final day of data collection to directly ask people whether they had received the bivalent booster. This question was presented at the end of the survey, along with a question asking them whether they had completed the primary COVID-19 vaccine series (we guaranteed participants that their responses would not affect their payment). For people who participated in the study on the fourth day, those who had not completed the primary series or who already received the bivalent booster were excluded from our analysis.

Following our preregistration, we first compared the three information-provision messages to determine whether we should collapse them into one condition. We predicted Perceived Persuasiveness and Booster Intentions using OLS regressions, with an indicator of the Info-Uniqueness Message and an indicator for the Info-Severity message (with the Info-Eligibility Clarification message as the reference group). These regressions only included observations from these three information-provision conditions. When the outcome measure was Perceived Persuasiveness (Supplementary Table 3 Column 1), a joint significance F-test was significant ($F(2, 884) = 5.78$, $p = 0.003$), rejecting the null hypothesis that the three information-provision messages had the same true value of Perceived Persuasiveness. However, when the outcome measure was Booster Intentions (Supplementary Table 3 Column 4), a joint significance F-test of the coefficient on Info-Uniqueness and the coefficient on Info-Severity was not significant ($F(2, 884) = 1.89$, $p = 0.15$), failing to reject the null hypothesis that the three message conditions had the same true value of Booster Intentions. In light of the inconclusive evidence for whether or not the three information-provision messages differed from each other, we ran two sets of analyses to compare these messages with the Simple-No Info message.

In one set of OLS regressions, we combined the three information provision messages into one aggregate group. That is, we predicted Perceived Persuasiveness and Booster Intentions as a function of three key predictors: (1) a binary indicator, Information Provision, which equals one if a patient received one of the three information-provision messages and zero if the patient received the Simple-No Info message; (2) a binary indicator for getting the Consistency message; and (3) a binary indicator for getting the Consistency & Info-Uniqueness message. The Simple-No Info message was the reference group in these regressions. The results are reported in Supplementary Table 3 Columns 2 and 5.

In another set of regressions, we separately compared each message with the Simple-No Info message. That is, we predicted Perceived Persuasiveness and Booster Intentions with five binary indicators of the five treatment messages (with the Simple-No Info message as the reference group). The results are shown in Supplementary Table 3 Columns 3 and 6.

We next explain two deviations from the preregistration. First, we had initially preregistered to collect 3,600 responses and test another version of the text messages that did not mention booster appointments were limited at the clinics. However, after the first day of data collection, we realized that the rate at which people met our selection criteria was much lower than our expectation. Out of 1,185 workers who responded, only 560 (47%) had completed the COVID-19 vaccine primary series and had not received any COVID-19 vaccine dose within the past two months. This made it challenging for us to recruit 3,600 participants, which would have required nearly 8,000 MTurk workers to respond to our survey. Additionally, the version of text messages that did not mention the limited booster appointments at the clinics may seem odd to participants, as participants may wonder why their healthcare providers would not offer bivalent boosters at their own clinics but instead encourage patients to get the booster at a local pharmacy. For these reasons, we dropped this version of text messages from the second day of data collection onwards and aimed to recruit 1,800 eligible participants to react to the version of text messages that mentioned limited booster appointments at the clinics[1], as the text messages in our RCTs did. Our final sample included 1,774 participants who met our aforementioned selection criteria. The final sample size was lower than 1,800 because as mentioned earlier, for the last (fourth) day of data collection, the questions we used to exclude people from analyses were presented at the end of the study. When enrolling participants on the last day, we could only estimate but not precisely predict how many participants who took the study that day would be excluded from the analysis.

Second, after we started data collection, we realized that we forgot to include questions to assess the psychological processes that may drive people to respond differently to the Consistency and Consistency & Info-Uniqueness messages versus the Simple-No Info message. We then added two questions for this purpose towards the end of the survey (after all preregistered mechanism questions, but before vaccination history and demographics questions). As a result, some participants in our dataset did not answer those two questions.

We also noted that unexpectedly, on the last day of data collection (the only day when we asked people to report at the end of the survey whether they had already received the bivalent booster, and used this question to filter people out of analyses), the proportion of people who

[1] For example, the Simple-No Info message that mentioned limited appointments read as, "[Your first name], you can now get the new bivalent COVID-19 booster. Our clinics have limited booster appointments available. Book your appointment at a Pharmacy nearby (more availability): [Link to locate pharmacies nearby]" The Simple-No Info message that did not mention limited appointments read as, "[Your first name], you can now get the new bivalent COVID-19 booster. Book your appointment at a Pharmacy nearby: [Link to locate pharmacies nearby]."

reported getting the booster was significantly higher in one condition (the Consistency & Info-Uniqueness condition) than in several other conditions. We view this as happening by chance because, in theory, the Consistency & Info-Uniqueness message could not change whether people had already received the booster. We conducted two robustness checks: (1) we included all participants who responded to our primary outcome measures on the last day of collection, regardless of whether they reported having already received the bivalent booster; and (2) we only examined participants who took our study during the first three days of data collection, when we screened out people who failed our selection criteria at the beginning of the survey and then randomized eligible participants into conditions. All of the key results hold for Perceived Persuasiveness and Booster Intentions, except that the difference between the Consistency message and the Simple-No Info message in Booster Intentions in the second robustness check becomes only significant at the 10% level (p = .06), though the regression coefficient is largely unchanged from the main analysis (i.e., from 0.385 to 0.370).

Online Studies Examining Messages Tested in RCT3 and Laypeople Predictions

Following our preregistration, we began by examining whether the two vaccine bundling messages worked differently from each other, in order to decide whether we should collapse them into one condition to be compared with the Simple-Enhance Protection message. In OLS regressions that only included participants assigned to the two vaccine bundling messages, we predicted Perceived Persuasiveness, Booster Intentions, and Perceived Convenience—which were preregistered primary outcome measures in at least one of the two experiments—with an indicator for the Bundle-Tagging Flu Shot message (with the reference group being the Bundle-Booster & Flu Shot message). As shown in Supplementary Table 5, the two bundling messages significantly differed in Perceived Persuasiveness (B = -0.353, p = 0.004, 95% CI = [-0.592, -0.115]) but did not have a detectable difference in Booster Intentions (B = 0.136, p = 0.380, 95% CI = [-0.168, 0.439]) or Perceived Convenience (B = 0.102, p = 0.44, 95% CI = [-0.156, 0.360]). In light of the inconclusive evidence for whether or not the two bundling messages differed from each other, we ran two sets of analyses to compare these messages with the Simple-Enhance Protection message.

In one set of OLS regressions that included all participants in the sample, we combined the two vaccine bundling messages to be compared with the Simple-Enhance Protection message. That is, we predicted Perceived Persuasiveness, Booster Intentions, and Perceived Convenience with a binary variable, Bundling, which equals one if participants read one of the two vaccine bundling messages and zero if participants read the Simple-Enhance Protection message. In another set of regressions, we separately compared each message with the Simple-Enhance Protection message. That is, we predicted Perceived Persuasiveness, Booster Intentions, and Perceived Convenience with two binary indicators of the two vaccine bundling messages (with the Simple-Enhance Protection message as the reference group). The results are shown in Supplementary Table 5.

In the Main Text we report prediction results using data from all 498 respondents who made a prediction. As a robustness check, here, we report results based on predictions made by the 363 respondents who reported that they completed the COVID-19 primary vaccine series and did not receive any COVID-19 vaccine dose in September, October, or November 2022—that is, people who met the basic eligibility for the COVID-19 bivalent booster at the time of our study. When asked to predict which message would yield the highest booster uptake rate, 71.90% of respondents predicted that the Bundle-Booster & Flu Shot message would be the most effective,

20.94% selected the Bundle-Tagging Flu Shot message, and the remaining 7.16% chose the Simple-Enhance Protection message. The proportion that picked the Bundle-Booster & Flu Shot message was significantly higher than the chance level of 33.3% ($z = 15.76$, $p < 0.001$). The proportion that selected either of the two bundling messages (i.e., either Bundle-Booster & Flu Shot or Bundle-Tagging Flu Shot) was significantly higher than the chance level of 66.6% ($z = 10.60$, $p < 0.001$).

We next describe sample size rationale and deviations from our preregistration of the first online experiment accompanying RCT3. For the first experiment, we estimated that we needed at least 176 participants per condition (i.e., 528 in total across three conditions) who satisfied our preregistered eligibility criteria, in order to detect an effect size of Cohen's $d = 0.3$ between two conditions with 80% statistical power for our main outcome variables. To have greater statistical power, we preregistered that we aimed to enroll 800 participants who satisfied our eligibility criteria. We originally did not plan to measure how convenient people felt it would be to get the flu shot at the same time as the booster (Perceived Convenience); thus, this variable did not appear in our preregistration. We realized after data collection had started that it would be useful to measure this construct, since the intention behind the bundling message was to make it seem convenient to get the flu shot at the same time as the booster. Thus, we added the measure of Perceived Convenience during the second day of our data collection, with the plan to report the addition of this variable as a deviation from the preregistration. However, we then felt that it would be the best to start from scratch and run a study that closely followed our preregistration. Thus, we stopped the first online experiment partially through the second day of data collection, before reaching the preregistered sample size. We then preregistered the second online experiment, included Perceived Convenience as one preregistered outcome measure, and conducted the second online experiment exactly following the preregistration. For the second experiment, we preregistered that we aimed to enroll 600 participants who satisfied our eligibility criteria. We explained in the preregistration that "Since we do not know in advance the percentage of participants that will satisfy our selection criteria, we will launch the study to more participants and the eventual number of participants that satisfy our criteria may not be exactly 600. Since we cannot exclude participants on Prolific based on these eligibility criteria, participants who do not satisfy these criteria will also respond to our survey."

Online Survey Exploring Beliefs Associated with Vaccination Intentions

To measure Booster Intentions, we asked participants whether they intended to get the bivalent booster, with 1 indicating "I have already gotten the COVID-19 bivalent booster", 2 indicating "I plan to get the COVID-19 bivalent booster but I have not done it yet", 3 indicating "I am not sure if I will get the COVID-19 bivalent booster", and 4 indicating "I do not plan to get the COVID-19 bivalent booster." The following belief and perception measures are particularly important to shaping the design of our text messages.

- Perceived Eligibility: Participants indicated whether they thought they were eligible to get the bivalent booster, with 1 equaling "Yes, I believe I am eligible", 2 equaling "I am not sure", and 3 equaling "No, I don't believe I am eligible." We reversely coded this variable so a higher value indicates a more certain belief that one was eligible for the bivalent booster. That is, in our analysis, 3 equals "Yes, I believe I am eligible", 2 equals "I am not sure", and 1 equals "No, I don't believe I am eligible."
- Infection Likelihood and Booster Effectiveness for Infections: Participants reported their likelihood of getting infected with the coronavirus if they did not receive the COVID-19

bivalent booster (Infection Likelihood without Booster) and if they did receive the COVID-19 bivalent booster (Infection Likelihood with Booster) (from 0% = I certainly won't get infected with COVID-19 to 100% = I certainly will get infected with COVID-19). For each participant, we subtracted Infection Likelihood with Booster from Infection Likelihood without Booster to capture their beliefs about the effectiveness of the COVID-19 booster in reducing their chance of getting infected with the coronavirus (Booster Effectiveness for Infections), which in theory could range from -100% to 100%.

- Long COVID Likelihood and Booster Effectiveness for Long COVID: After introducing Long COVID, which we defined as a range of health problems that can last weeks or even months after COVID-19 infections, we asked participants to report their likelihood of getting Long COVID if they unfortunately became infected with COVID-19. Participants reported the likelihood under two circumstances: One was when they did not receive the COVID-19 bivalent booster (Long COVID Likelihood without Booster), and the other was when they did receive the COVID-19 bivalent booster (Long COVID Likelihood with Booster) (from 0% = I certainly wouldn't develop Long COVID to 100% = I certainly would develop Long COVID). To measure beliefs about the effectiveness of the COVID-19 booster in combating Long COVID (Booster Effectiveness for Long COVID), we subtracted Long COVID Likelihood with Booster from Long COVID Likelihood without Booster.
- Infection Severity and Booster Effectiveness for Severe Symptoms: Participants also reported how ill they thought they would be if they unfortunately got infected with COVID-19 (from 0 = No symptoms to 10 = Severely ill/hospitalized), and they did so for two circumstances. One circumstance was when they did not receive the COVID-19 bivalent booster (Infection Severity without Booster) and the other was when they did receive the COVID-19 bivalent booster (Infection Severity with Booster). The difference between these two measures reflects participants' beliefs about the effectiveness of the COVID-19 booster in alleviating COVID-19 symptoms (Booster Effectiveness for Infection Severity).
- Comparative Efficacy over Original Booster: Participants rated the extent to which they agreed with the statement, "I believe the bivalent COVID-19 boosters are much more effective than the original COVID-19 boosters in protecting people against the dominant Omicron variants (BA.4 and BA.5)" (from 0 = Strongly disagree to 10 = Strongly agree).
- Guideline Confusion: Participants rated the extent to which they agreed with the statement, "I think the current public health guidelines about who should get the bivalent booster are confusing" (from 0 = Strongly disagree to 10 = Strongly agree).
- Perceived Doctor Recommendation: Participants predicted, "What percentage of doctors do you think are recommending their eligible patients to get the bivalent COVID-19 booster?" (from 0% to 100%).

Across the 533 survey respondents, 10.13% reported having already received the bivalent booster, 54.03% reported that they planned to get it but had not done so yet, 27.95% were unsure about getting the bivalent booster, and the remaining 7.88% did not plan to get it.

To inform the design of text messages, we compared beliefs about the coronavirus and the bivalent booster across participants based on whether they planned to get the booster (including those who already got it and those who had not received it), were uncertain, or did not plan to get the booster. Supplementary Table 9 displays the mean and standard deviation of the

key measures (highlighted above) for the three sub-groups of participants. We also report the p-values of two-tailed t-tests that compare people who planned to get the booster with those who felt uncertain or did not plan to get it, along with the 95% confident intervals of the differences and Cohen's *d*s. Based on the skewness and kurtosis test for normality, all of our key measures violate the normality assumption, except for Infection Severity without Booster. Also, apart from Long Covid Likelihood without Booster and Infection Severity without Booster, all other key measures violate the equal variance assumption. Our reported results in Supplementary Table 9 are robust to using non-parametric Mann-Whitney U-tests to compare people who planned to get the booster with those who felt uncertain or did not plan to get it.

To summarize the key take-aways, we find that compared to those who felt uncertain or did not intend to get the bivalent booster, people who planned to get the booster:

- Were more likely to realize that they were eligible for the bivalent booster.[2] In fact, people who indicated uncertainty about booster intentions were less aware of their eligibility ($M = 2.50$, $SD = 0.50$), compared to both participants who intended to get the bivalent booster ($M = 2.80$, $SD = 0.43$, $t(489) = 6.78$, $p < 0.001$) and those who did not intend to get the booster ($M = 2.74$, $SD = 0.45$, $t(189) = 2.82$, $p < 0.001$). A closer look at the data suggests that among people who intended to get the booster, a large portion (80.99%) knew they were eligible; and a similarly large (73.81%) of participants who did not plan to get the booster knew they were eligible. In contrast, only 49.66% of people with uncertain booster intentions knew they were eligible, and the remaining 50.34% said they did not know if they were eligible.
- Believed that if they did not get the bivalent booster, they would have a greater likelihood of getting infected with COVID-19, a higher likelihood of developing Long COVID, and more severe COVID-19 symptoms.
- Believed more strongly in the effectiveness of the bivalent booster in reducing the likelihood of getting infected with COVID-19, the chance of developing Long COVID, and the severity of COVID-19 symptoms
- Believed more strongly that the bivalent booster offered greater protection over the dominant Omicron variants than the original boosters
- Expressed less confusion about public health guidelines on the bivalent booster
- Believed that a greater percentage of doctors recommended the bivalent booster

Supplementary Notes

Booster Vaccination Location

As explained in Methods, from the fourth day of our RCTs forward, we only included one link in each message (with the exception of the Doctor Recommendation Only arm, which had no links), either directing patients to CVS Pharmacy or directing them to a general website

[2] At the time of our study, CDC guidelines indicated that adults who had completed the COVID-19 primary vaccine series and had not received a vaccine dose within two months were eligible for the bivalent booster. The guidelines also stated that people who had recently contracted COVID-19 may consider delaying getting a vaccine dose for three months (https://www.cdc.gov/coronavirus/2019-ncov/vaccines/stay-up-to-date.html). Our finding about the difference in eligibility awareness between subgroups of participants holds, when we exclude those who had received the bivalent booster, contracted COVID-19 in the past 90 days, or received a vaccine dose within the past two months.

with many vaccine venues depending on their condition. To understand whether and how patients' likelihood of getting the bivalent booster at CVS Pharmacy varied between those who were prompted to consider specific venues and those who were prompted to search among many vaccine venues, we focus on patients who were enrolled in our RCTs from the fourth day forward. This gives us a clean comparison, as those patients only got one link in the message (either a narrow link to CVS Pharmacy or a broad link to a general website).

Among patients in our analysis sample who were enrolled from the fourth day forward, received a narrow link to CVS Pharmacy, and got the bivalent booster within 4 weeks of the assigned message date, 48.23% got the booster from CVS Pharmacy. That rate was statistically significantly higher than the proportion observed among patients whose message included a link to a general website with many vaccine venues (i.e., 44.69%; $\mathcal{X}^2(1) = 27.41$, $p < 0.001$). This observation suggests that a) the inclusion of a link to a specific venue attracted more patients to get the booster at the specific venue and b) CVS Pharmacy was already a popular venue for patients to obtain the COVID-19 bivalent booster as almost half of patients who got the bivalent booster during our preregistered observation time window went to CVS Pharmacy even if they received a broad link to a general website where they could find other vaccine venues.[3]

Effect Size Comparison with Previous Field Work in the Same Population

One of the outcome measures in Saccardo et al. (2023) is comparable to ours: the take-up of the first COVID-19 vaccine dose anywhere in California within four weeks. Using this outcome measure, Saccardo et al. (2023) estimated that receiving (vs. not receiving) a reminder in their first randomized controlled trial on average increased the initial COVID-19 vaccine uptake by 1.0 percentage point, and that adding ownership language increased the first dose uptake by 0.6 percentage points (relative to the reminders without such language). These effect sizes are similar to what we find here: receiving one reminder on average increased booster uptake in California within four weeks by 1.13 percentage points, and the addition of ownership framing is estimated to increase the booster uptake rate by approximately 0.8 percentage points, relative to the messages without this language.

Examples of Text Messages Related to COVID-19 Vaccinations

1. Messages from the City of Los Angeles in 2021 to promote COVID-19 vaccinations

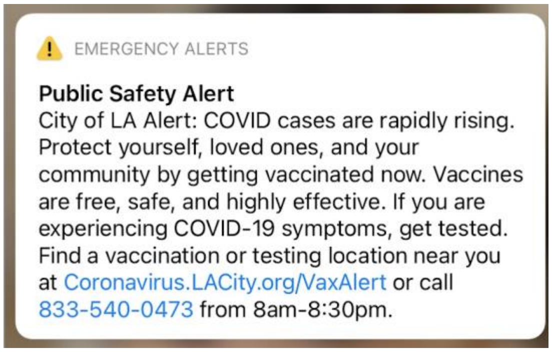

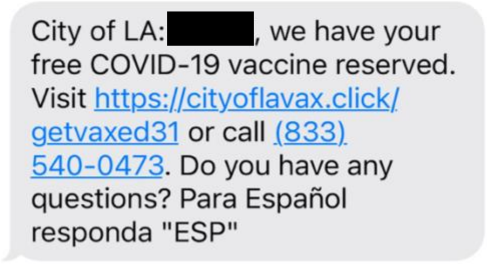

[3] In case readers are interested in knowing about the popularity of UCLA Health as a booster venue, we also provide information about the proportion of patients who received the booster from either CVS Pharmacy or UCLA Health. The percentage was 57.90% among patients receiving a narrow link to CVS Pharmacy versus 54.12% among patients receiving a broad link ($\mathcal{X}^2(1) = 31.50$, $p < 0.001$).

2. Messages from Walgreens and CVS Pharmacy to promote the uptake of bivalent booster and/or flu shot, some of which encouraged customers to bundle flu shot and COVID-19 booster and some of which contained the ownership language.

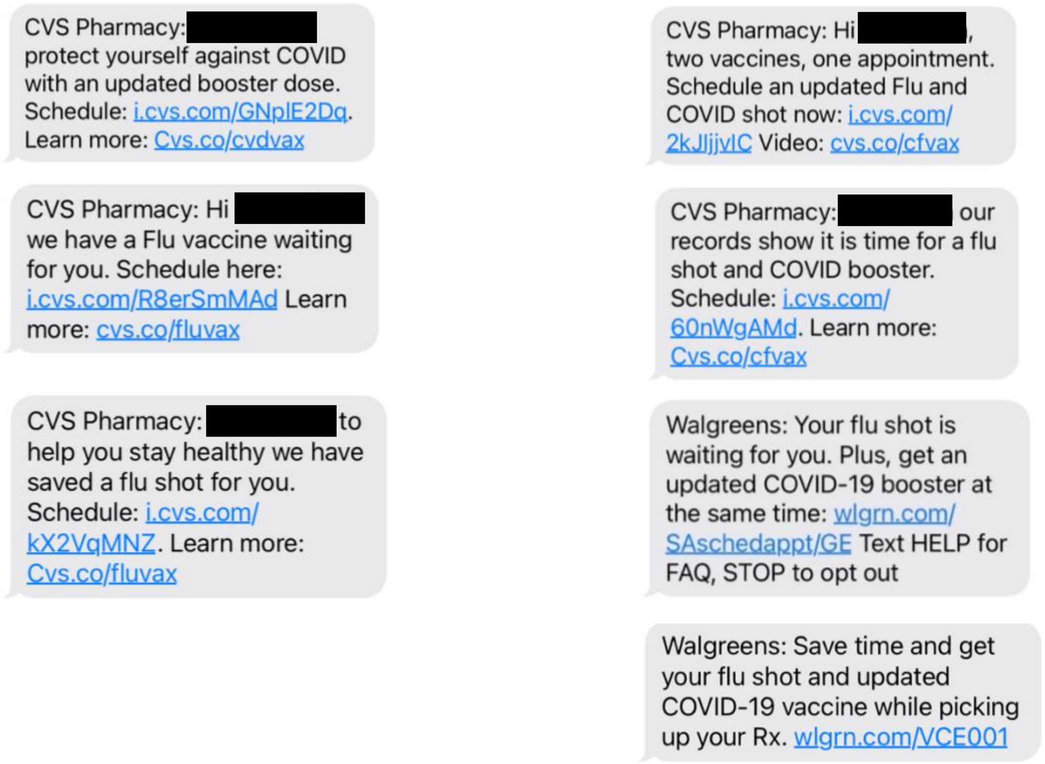

Note: Images presented in this section are screenshots taken by the authors of text messages sent to them.
