# Supplementary material for: Field testing the transferability of behavioural science knowledge on promoting vaccinations
Source: Nat Hum Behav. 2024 Mar 14;8(5):878–90. doi: 10.1038/s41562-023-01813-4 (PMC11132983; doi:10.1038/s41562-023-01813-4)
Supplement: Supplementary file 2 — Reporting Summary [file 41562_2023_1813_MOESM2_ESM.pdf]

nature portfolio

Corresponding author(s): Silvia Saccardo

Last updated by author(s): Dec 8, 2023

Reporting Summary

Nature Portfolio wishes to improve the reproducibility of the work that we publish. This form provides structure for consistency and transparency in reporting. For further information on Nature Portfolio policies, see our Editorial Policies and the Editorial Policy Checklist.

Statistics

For all statistical analyses, confirm that the following items are present in the figure legend, table legend, main text, or Methods section.

n/a	Confirmed	
☐	☒	The exact sample size (n) for each experimental group/condition, given as a discrete number and unit of measurement
☐	☒	A statement on whether measurements were taken from distinct samples or whether the same sample was measured repeatedly
☐	☒	The statistical test(s) used AND whether they are one- or two-sided<br>*Only common tests should be described solely by name; describe more complex techniques in the Methods section.*
☐	☒	A description of all covariates tested
☐	☒	A description of any assumptions or corrections, such as tests of normality and adjustment for multiple comparisons
☐	☒	A full description of the statistical parameters including central tendency (e.g. means) or other basic estimates (e.g. regression coefficient) AND variation (e.g. standard deviation) or associated estimates of uncertainty (e.g. confidence intervals)
☐	☒	For null hypothesis testing, the test statistic (e.g. F, t, r) with confidence intervals, effect sizes, degrees of freedom and P value noted<br>*Give P values as exact values whenever suitable.*
☒	☐	For Bayesian analysis, information on the choice of priors and Markov chain Monte Carlo settings
☒	☐	For hierarchical and complex designs, identification of the appropriate level for tests and full reporting of outcomes
☐	☒	Estimates of effect sizes (e.g. Cohen's d, Pearson's r), indicating how they were calculated

Our web collection on statistics for biologists contains articles on many of the points above.

Software and code

Policy information about availability of computer code

Data collection	Data about randomized controlled trials (RCTs) were extracted from the medical records of UCLA Health patients by a UCLA Health analyst. Data from all online and prediction surveys were collected via Qualtrics.
Data analysis	Data analysis was conducted in Stata 14. The code to replicate the analyses and figures in the Main Text and the Supplementary Information is available at https://osf.io/qhw95.

For manuscripts utilizing custom algorithms or software that are central to the research but not yet described in published literature, software must be made available to editors and reviewers. We strongly encourage code deposition in a community repository (e.g. GitHub). See the Nature Portfolio guidelines for submitting code & software for further information.

Data

Policy information about availability of data

All manuscripts must include a data availability statement. This statement should provide the following information, where applicable:

- Accession codes, unique identifiers, or web links for publicly available datasets
- A description of any restrictions on data availability
- For clinical datasets or third party data, please ensure that the statement adheres to our policy

The data analyzed in this article about randomized controlled trials were provided by UCLA Health and contain protected health information. To protect participant privacy, we cannot publicly post individual-level data. Upon request to the corresponding authors, and approval by the UCLA Health Data Oversight committee,

qualified researchers can obtain access to the deidentified data about these trials. A formal contract will be signed and an independent data protection agency should oversee the sharing process to ensure the safety of the data. Data about all our surveys are available at https://osf.io/qhw95.

Research involving human participants, their data, or biological material

Policy information about studies with human participants or human data. See also policy information about sex, gender (identity/presentation), and sexual orientation and race, ethnicity and racism.

Reporting on sex and gender	All regressions, unless otherwise explained, control for patient gender (male, female, with people whose gender was "other" or unknown to us as the reference group), as pre-registered. In the RCTs, patients' gender was obtained from patients' medical record. In the online studies, participants' gender was self-reported.
Reporting on race, ethnicity, or other socially relevant groupings	All regressions for the RCTs, unless otherwise explained, control for race/ethnicity, using the following categories: Hispanic, White non-Hispanic, Black non-Hispanic, Asian non-Hispanic, other/mixed race, with people whose race was unknown to us and whose ethnicity was not Hispanic as the reference group, as pre-registered. All regressions for the online experiments, unless otherwise explained, control for race/ethnicity, using the following categories: Hispanic, White non-Hispanic, Black non-Hispanic, Asian non-Hispanic, with people whose race was other or mixed or unknown to us and whose ethnicity was not Hispanic as the reference group. In the RCTs, data on patients' race/ethnicity were obtained from patients' medical record. In the online studies, participants' race/ethnicity was self-reported.
Population characteristics	See Main Text, Methods, and Extended Data Table 1 for detailed participant characteristics.
Recruitment	Our RCTs are part of the vaccination outreach effort at UCLA Health. All patients who fit our enrollment inclusion/exclusion criteria were automatically enrolled and randomized to condition (as well as one of the message dates and one of the three time slots in a given day). Since patients could not withdraw from the RCTs, no patient was lost to follow up, and treatment could not affect the inclusion/exclusion criteria for our analysis sample, randomization and causal inference are maintained for our analysis sample. Self-selection is not an issue in our case.<br><br>For the online experiments accompanying the second and third RCTs, participants were recruited on MTurk via CloudResearch and/or Prolific in exchange for a compensation. Regarding self-selection bias, due to informed consent procedures and the use of brief advertisements, people may have chosen to participate based on their knowledge of or interest in our survey topic. This is true for any survey study that involves participant consent. Because participants were randomly assigned to condition, it is unlikely self-selection would result in the effects observed in our online experiments.<br><br>For the layperson prediction component that was at the end of one experiment as well as for the survey assessing beliefs associated with booster uptake intentions, due to informed consent procedures and the use of brief advertisements, people may have chosen to participate based on their knowledge of or interest in our survey topic. This is true for any survey study that involves participant consent.<br><br>For the expert prediction survey, we invited attendees of two conferences, the 2022 Annual Behavioral Economics and Health Symposium (November 10, 2022) and the 2022 Society of Judgment and Decision Making annual meeting (November 11-13, 2022), to participate in a brief survey. There was no compensation involved. This is a selective sample involving only behavioral scientists who were willing to volunteer their time to take a prediction survey for free. But this bias is likely to exist for any free prediction survey involving experts.
Ethics oversight	This research was deemed to comply with all relevant ethical regulations. The Institutional Review Board at the University of California Los Angeles approved the protocols of our randomized controlled trials (reference number 21-000268) and determined that a waiver of informed consent was appropriate. The online experiments and the vaccination intention survey were conducted under approval of the Institutional Review Board at Carnegie Mellon University (reference number STUDY2020_00000347), and informed consent was obtained from online study participants as part of the enrollment process. The expert prediction survey was deemed as non-human subject research by the Institutional Review Board at the University of California Los Angeles.

Note that full information on the approval of the study protocol must also be provided in the manuscript.

Field-specific reporting

Please select the one below that is the best fit for your research. If you are not sure, read the appropriate sections before making your selection.

☐ Life sciences ☒ Behavioural & social sciences ☐ Ecological, evolutionary & environmental sciences

For a reference copy of the document with all sections, see nature.com/documents/nr-reporting-summary-flat.pdf

Behavioural & social sciences study design

All studies must disclose on these points even when the disclosure is negative.

Study description	In three RCTs, we varied whether patients received a text message as well as the type of message they got, and assessed whether they subsequently obtained the COVID-19 booster. In three online experiments, we presented participants with one of the text

messages used in our second or third RCT, and assessed their perceived persuasiveness of the message and/or their interest in getting the COVID-19 booster along with other measures. These data are all quantitative experimental. In two prediction surveys, we presented behavioral scientists or online participants three messages used in the third RCT and asked them to predict which message would work the best. The laypeople prediction component was placed at the end of one experiment.

Research sample

For the RCTs, our analysis includes 314,824 UCLA Health patients who satisfied all the preregistered inclusion and exclusion criteria described in the Methods (e.g., eligible for the bivalent booster but having not already received it before the message date). Those patients were, on average, 49.96 years old (SD = 17.80), 42.20% were male, 48.76% were White (excluding Hispanic patients), and 14.21% were Hispanic (see Extended Data Table 1 for demographics by condition and balance checks). We chose UCLA Health patients as our study sample because UCLA Health is one of the largest healthcare systems in California (which allows us to assess a large patient population) and was supportive of evaluating the effectiveness of behavioral interventions in promoting COVID-19 bivalent booster uptake. Our RCT sample is not representative.

For the experiment accompanying RCT2, a total of 1,774 participants met our selection criteria described in the Methods, responded to our preregistered outcome measures, and were thus included in our analysis. They were an average of 42.06 years old (SD=12.92), 47.69% were male, 73.56% were White (excluding Hispanic participants), and 4.28% were Hispanic.

Across the two experiments accompanying RCT3, a total of 989 participants met our selection criteria described in the Methods, and responded to Perceived Persuasiveness (the preregistered outcome measure in both experiments). These participants had an average age of 34.78 years old (SD=12.80), 48.03% were male, 68.15% were White (excluding Hispanic participants), and 6.27% were Hispanic.

For the survey assessing beliefs associated with booster uptake intentions, we recruited 533 adults from Prolific (n=349) and MTurk via CloudResearch (n=184) who had completed the COVID-19 primary vaccine series, lived in California, passed an attention check (for MTurk only), and finished the survey. They were, on average, 37.57 years old (SD=13.46), 56.29% were male, 48.78% were White (excluding Hispanic participants), 13.13% were Hispanic, and 97.19% were living in California at the time of the study.

For all of our online surveys, the samples are not representative and are “convenience samples” from online survey platforms that social scientists commonly use.

A total of 40 conference attendees responded to our expert prediction survey during two conferences, comprising 47.5% faculty members and 45% post-docs, PhD students, or other academic positions. We were interested in behavioral scientists' predictions, and those conferences took place right after our trials had concluded but before we had access to data. Thus, these conferences presented great opportunities for data collection.

Sampling strategy

Our goal was to include in the study any UCLA Health patients who were eligible for the COVID-19 bivalent booster and could be reached out via SMS message. For this reason, our initial enrollment criteria include UCLA Health primary care or specialty attributed patients who: (1) completed the COVID-19 primary vaccine series as of October 10, 2022 based on the most comprehensive immunization records UCLA Health could access at that time, (2) did not receive any COVID-19 dose within two months prior to October 10, 2022, (3) were at least 18 years old, and (4) had a phone number on file that had not previously been opted out of UCLA Health text messaging. This initial eligibility determination process resulted in 386,615 patients. We obtained this list on October 10, 2022, and enrolled all of these patients in our RCTs. However, since vaccination records get updated over time and some patients may get the bivalent booster between October 10, 2022 and their assigned message date, we preregistered to exclude from our analysis patients who received any dose of COVID-19 vaccine within the 2 months before their assigned message date, based on the administrative records obtained at the time of final data collection. Due to this exclusion criteria, the exact sample size could not be determined before the RCTs started. Based on conversations with UCLA Health, we expected to have at least 150,000 patients in the final analysis sample across the three RCTs, so we knew we had at least 80% statistical power to detect about a 1-percentage-point difference between the Holdout arm and the text message arms combined, assuming that the Holdout arm would have a baseline take-up rate of 50% (two-sided proportion test, alpha = 0.05). This sample is convenience based.

For all the online surveys on Prolific and MTurk, the sampling procedure was convenience based, and participants opted into our studies after reading our recruitment materials on Prolific or MTurk. For the online experiment accompanying RCT2, we aimed to obtain 1,800 participants in total across six conditions, in order to have 80% statistical power to detect differences of a small magnitude (Cohen’s d around 0.25) between the Simple-No Info message and each of the other treatment messages. For the first and second online experiments accompanying RCT3, we aimed to obtain 800 and 600 participants, respectively, in order to have at least 80% statistical power to detect an effect size of Cohen's d of 0.3 between the Simple-Enhance Protection message and each of the other two treatment messages. See Supplementary Methods for deviation from the planned sample size in the first online experiment accompanying RCT3.

For the expert prediction survey, we enrolled as many experts as we could at the two aforementioned conferences. This is a convenience sample.

Data collection

For the RCTs, data were extracted from medical records of UCLA Health patients by a UCLA Health analyst who was blind to condition and hypothesis; enrollment and text message delivery were implemented by UCLA Health and a messaging vendor who were blind to the hypotheses. For online experiments, data were collected electronically via Qualtrics, which completed the randomization into separate experimental conditions; researchers were blind to experimental condition at the data collection stage. For expert prediction survey and the survey assessing beliefs associated with booster uptake intentions, data were collected electronically via Qualtrics.

Timing

Participants for the RCTs were enrolled during 11 work days from October 18, 2022 to November 1, 2022. Data on vaccination records and participant characteristics were extracted on July 7, 2023. Data for the online experiment accompanying the second RCT were collected on October 25-28, 2022. Data for the online experiments accompanying the third RCT were collected on November 4, 7, 14, and 15, 2022. For the behavioral scientist prediction survey, data were collected during two conferences (from November 10, 2022 to November 13, 2022). Data for the survey assessing beliefs associated with booster uptake intentions were collected on September 16, 2022.

Data exclusions	Following our preregistration, at the analysis stage, we exclude 71,791 patients from the three RCTs. First, our analyses exclude patients who received any dose of COVID-19 vaccine within the two months (or precisely 60 days) before their assigned message date, because those patients were not eligible for receiving the bivalent booster at the time of getting our message. Though we already tried our best to take into account whether patients received a dose within two months before October 10, 2022 (i.e., when we selected the pool of patients to enroll), vaccination records get updated over time, and some patients may get a dose between October 10, 2022 and their assigned message date.<br><br>Further, we exclude patients who, as far as UCLA Health could track, received the COVID-19 bivalent booster before the assigned message date, or had died before the study. We also preregistered that we would exclude patients who scheduled a booster appointment at UCLA Health before the assigned message time. However, we ended up not using the data about appointments for two reasons. One is that the staff shortage at UCLA Health meant that only a limited number of patients were able to schedule bivalent booster appointments there. The other reason is that we learned after the RCTs ended that some patients were able to get a bivalent booster at a doctor visit during our experiment period without making a bivalent appointment, which means that before their assigned message time, some patients may have already planned to get the booster at their upcoming normal doctor appointments but we could not tell it from the bivalent booster appointment data.
Non-participation	For the RCTs, all participants who fit our eligibility criteria were automatically enrolled, and nobody actively dropped out. For our surveys, no participants requested to withdraw their responses.
Randomization	For the three RCTs, patients were first randomly assigned to either one of the 14 message conditions or the holdout condition. The chance of being randomly assigned to any given message condition was the same across the 14 messages conditions, and the chance of being randomly assigned to the holdout condition was three times the chance of being randomly assigned to one message condition. We oversampled the holdout condition because we had three parallel RCTs. Within each condition, patients were then randomly assigned to one of 11 workdays (from October 18, 2022 to November 1, 2022) and one of three time slots (9am, 12pm, and 4pm), which allowed us to keep the total number of messages sent at any given point in time within the limit imposed by UCLA Health's text messaging vendor. See Methods for details.<br><br>Participants in the online experiments were randomly assigned with an equal probability to read one of the text messages from our second or third RCT. In the prediction surveys, no random assignment was involved as participants were presented with three messages to pick. In the survey assessing beliefs associated with booster uptake intentions, no randomization was involved.

Reporting for specific materials, systems and methods

We require information from authors about some types of materials, experimental systems and methods used in many studies. Here, indicate whether each material, system or method listed is relevant to your study. If you are not sure if a list item applies to your research, read the appropriate section before selecting a response.

Materials & experimental systems

n/a	Involved in the study
☒	☐ Antibodies
☒	☐ Eukaryotic cell lines
☒	☐ Palaeontology and archaeology
☒	☐ Animals and other organisms
☐	☒ Clinical data
☒	☐ Dual use research of concern
☒	☐ Plants

Methods

n/a	Involved in the study
☒	☐ ChIP-seq
☒	☐ Flow cytometry
☒	☐ MRI-based neuroimaging

Clinical data

Policy information about clinical studies
All manuscripts should comply with the ICMJE guidelines for publication of clinical research and a completed CONSORT checklist must be included with all submissions.

Clinical trial registration	RCT1, https://clinicaltrials.gov/ct2/show/NCT05586204; RCT2, https://clinicaltrials.gov/ct2/show/NCT05586178; RCT3, https://clinicaltrials.gov/ct2/show/NCT05586165
Study protocol	Detail about the full trial protocol (the exact content of text messages) is available in Main Text (particularly Table 1) and at https://osf.io/qhw95.
Data collection	The RCTs were ran on 11 workdays from October 18, 2022 to November 1, 2022. The data reported in the paper were extracted from UCLA Health patients' medical records by a UCLA Health analyst on July 7, 2023. The observation window for our preregistered primary outcome was four weeks of the assigned message date (i.e., up to November 28, 2022).
Outcomes	We extracted patients' vaccination records from the California Immunization Registry (CAIR), which is the most comprehensive database for tracking vaccinations obtained across pharmacies and health clinics in California (including UCLA Health), and we complemented CAIR with Epic' interoperability platform to additionally capture vaccinations occurred outside California documented in patients' electronic health records. As preregistered, our primary outcome measure is a binary indicator of whether patients obtained a COVID-19 bivalent booster within four weeks of their assigned message date.
